# PRIZM: combining low-N data and zero-shot models to design enhanced protein variants

**DOI:** 10.1093/bioadv/vbag299

**Published:** 2026-10-06

**Authors:** David Harding-Larsen, Brianna M Lax, Martina Escorial García, Catarina Mendonça, Felipe Mejia-Otalvaro, Ditte Hededam Welner, Stanislav Mazurenko

**Affiliations:** The Novo Nordisk Foundation Biotechnology Research Institute for the Green Transition, Technical University of Denmark, DK-2800 Kongens Lyngby, Denmark; The Novo Nordisk Foundation Biotechnology Research Institute for the Green Transition, Technical University of Denmark, DK-2800 Kongens Lyngby, Denmark; The Novo Nordisk Foundation Biotechnology Research Institute for the Green Transition, Technical University of Denmark, DK-2800 Kongens Lyngby, Denmark; The Novo Nordisk Foundation Biotechnology Research Institute for the Green Transition, Technical University of Denmark, DK-2800 Kongens Lyngby, Denmark; The Novo Nordisk Foundation Biotechnology Research Institute for the Green Transition, Technical University of Denmark, DK-2800 Kongens Lyngby, Denmark; The Novo Nordisk Foundation Biotechnology Research Institute for the Green Transition, Technical University of Denmark, DK-2800 Kongens Lyngby, Denmark; Loschmidt Laboratories, Department of Experimental Biology and RECETOX, Faculty of Science, Masaryk University, Brno 625 00, Czech Republic; International Clinical Research Center, St. Anne’s University Hospital, Brno 602 00, Czech Republic

## Abstract

**Motivation:**

Machine learning has repeatedly shown the ability to accelerate protein engineering, but many approaches demand large amounts of robust, high-quality training data as well as substantial computational expertise. While large pre-trained models can function as zero-shot proxies for predicting variant effects, selecting the best model for a given protein property is often non-trivial.

**Results:**

Here, we introduce Protein Ranking using Informed Zero-shot Modelling (PRIZM), a two-phase workflow that first uses a high-quality low-N dataset to identify the most suitable pre-trained zero-shot model for a target protein property and then applies that model to rank and prioritize an *in silico* variant library for experimental testing. Across diverse benchmark datasets spanning multiple protein properties, PRIZM reliably separated low- from high-performing models using datasets of ∼20 labelled variants. We further demonstrate PRIZM in enzyme engineering case studies targeting sucrose synthase thermostability and glycosyltransferase activity, where PRIZM-guided selection identified improved variants, including gains of ∼3°C in apparent melting temperature and ∼20% higher relative activity. PRIZM provides an accessible, data-efficient route to leverage foundation models for protein design while requiring minimal experimental data.

**Availability and implementation:**

PRIZM is publicly available via Zenodo, DOI: 10.5281/zenodo.23035983, and the PRIZM GitHub repository https://github.com/daha-la/PRIZM.

## Graphical Abstract

**Figure:**
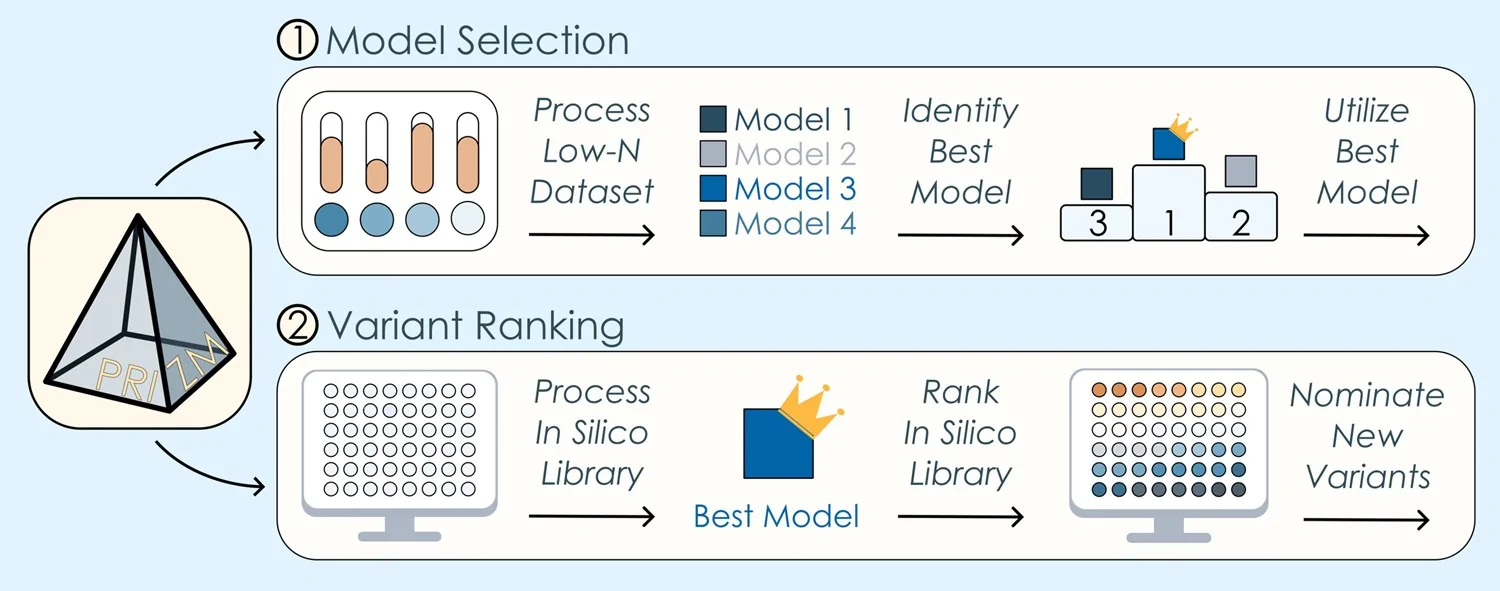

## 1 Introduction

Protein engineering encompasses the design and optimization of proteins with improved or novel functions by altering their amino acid sequences. Two predominant strategies guide this process: rational design, which relies on structural and mechanistic insight, and directed evolution, which utilizes iterative cycles of mutation and selection to explore sequence space empirically (Packer and Liu 2015, Arnold 2018, Lutz and Iamurri 2018). In recent years, machine learning (ML) has emerged as a complementary approach, enabling data-driven modelling of protein sequence–function relationships (Yang *et al.* 2019, Mazurenko *et al.* 2020, Braun *et al.* 2023). ML offers the potential to generalize beyond traditional domain knowledge, such as the structural and mechanistic understanding of how substitutions affect protein stability and activity, including at positions distant from functional sites (Tee *et al.* 2021, Raechell *et al.* 2026), while still leveraging available experimental and structural data.

ML-assisted directed evolution (MLDE) has shown impressive potential for efficiently traversing the fitness landscape of proteins, both reducing experimental demand and resulting in enhanced functionality (Yang *et al.* 2019). In MLDE, a supervised ML-based model is trained on an initial set of labelled variants and then used to nominate new variants for experimental characterization. Several strategies have been developed to make this viable at the low-N limit, including fine-tuning a language model on the target family before fitting a simple top model (Biswas *et al.* 2021), combining evolutionary and assay-labelled data in a single fitness model (Hsu *et al.* 2022a), parameter-efficient fine-tuning (Zhou *et al.* 2024), and in-context learning with test-time training (Teufel *et al.* 2025). The resulting data are iteratively fed back into the model, progressively improving its predictions. Recent MLDE methods leverage embeddings from large protein foundation models, ML-based tools trained on millions of natural sequences and structures using unsupervised objectives (Harding-Larsen *et al.* 2024a). This enables the models to generalize beyond the limited size of experimental datasets (Harding-Larsen *et al.* 2024a). In particular, the recently published EVOLVEpro workflow by Jiang *et al.* shows promise, drastically improving the hit rates of high-activity candidates using fewer than ten training rounds with only 16 mutants (Jiang *et al.* 2025).

In parallel to producing useful embeddings, large protein foundation models are used in a different mode. Because these models learn the constraints that govern natural protein families, they can provide meaningful estimates of how individual mutations affect protein properties even without an initial set of labelled variants, unlike their supervised counterparts. These models can thus be used in a zero-shot setting, where their internal scores serve as proxies for mutational effects without any task-specific training data. An example is the work by Hie *et al.*, where the authors developed a workflow that nominates new candidates by selecting variants predicted to outperform the wildtype (WT) protein according to multiple zero-shot models (Hie *et al.* 2024). Zero-shot models have also been used in smart variant library design, allowing researchers to make a preliminary *in silico* screening to enrich the initial pool (Wittmann *et al.* 2021).

While both supervised learning and zero-shot modelling have their benefits, both methodologies also have their own set of challenges—especially for non-experts. For supervised learning using small datasets, the most significant challenge is the susceptibility to overfitting. A robust and reliable train-test split is effectively impossible at the low-N limit, as neither subset is large enough to be statistically representative of the overall experimental dataset. To mitigate the risk, many state-of-the-art tools, such as the EVOLVEpro (Jiang *et al.* 2025), emphasize the creation of new, unbiased datasets to train robust and accurate models. However, this approach misses the opportunity to learn from existing experimental datasets, which ultimately increases experimental load and forgoes previously published data. Moreover, since supervised models are inherently tailored to specific engineering tasks, any transfer to different proteins or properties often requires model re-designing, retraining, and optimization (Biswas *et al.* 2021), creating the need for a ML expert in the team. For example, how to select the most suitable pre-trained protein embedding is still an open question (Harding-Larsen *et al.* 2024a). While benchmarking suites exist [e.g. TAPE (Rao *et al.* 2019)], many application papers still perform only *ad hoc* comparisons across a small number of models, and broad, systematic evaluations across the rapidly expanding set of models remain uncommon. In summary, this strong emphasis on careful model design and setup can make supervised MLDE daunting, if not downright impossible to implement for non-experts.

While zero-shot modelling appears easier to implement, it introduces its own challenges. Many models are available for zero-shot predictions, and this abundance can be a double-edged sword that makes choosing the most suitable model for a given engineering task a non-trivial challenge. Several studies have benchmarked zero-shot models against collections of deep mutational scan (DMS) datasets (Meier *et al.* 2021, Wittmann *et al.* 2021, Notin *et al.* 2023, Li *et al.* 2024b), with ProteinGym by Notin *et al.* being the most comprehensive to date (Notin *et al.* 2023). However, these benchmarks are often summarized as global averages across many proteins, which do not necessarily reflect performance on the specific protein or property of interest (Hsu *et al.* 2022b, Notin *et al.* 2023). When evaluated on subsets of tasks or proteins, the ranking of the models changes dramatically, implying that the best models may be task-specific. Such stratified analyses can themselves guide model choice, whether by assay type, MSA depth, taxon, and mutation depth (Notin *et al.* 2023), by assay features (Gurev *et al.* 2026), or by whether a property is better captured by stability- or conservation-based scores (Høie *et al.* 2022), though these analyses often narrow the choice rather than resolve it. Furthermore, zero-shot settings, by definition, do not incorporate any prior experimental data, which prevents models from adapting to specific systems or engineering objectives. As a result, model performance can vary between targets, and the best-performing model for one protein or protein property may be suboptimal for another.

Overall, these challenges highlight that neither supervised nor zero-shot approaches are straightforward for non-experts to apply in practice. Supervised methods demand careful model design and tuning, while zero-shot models offer no principled way to select the right predictor or adapt to the system at hand. This creates a need for an approach that combines the generality of foundation models with task-specific experimental insight, while avoiding specialized model setup. To address this need, we developed Protein Ranking using Informed Zero-shot Modelling (PRIZM). This novel ML approach combines the general domain knowledge of the large foundation models with the specialized insights obtained from low-N datasets to engineer enhanced protein variants. PRIZM consists of two phases: (1) Model Selection and (2) Variant Ranking (Fig. 1). In the first phase, the protein information—be it sequence, structure, or evolutionary patterns—is processed by a collection of pre-trained models (Table 1). The resulting zero-shot scores are then compared to an experimental low-N dataset to identify the model that best describes the targeted protein property. In the second phase, this best model is utilized to process and rank an *in silico* library, which serves as the basis for identifying new variants for experimental characterization.

**Figure 1:**
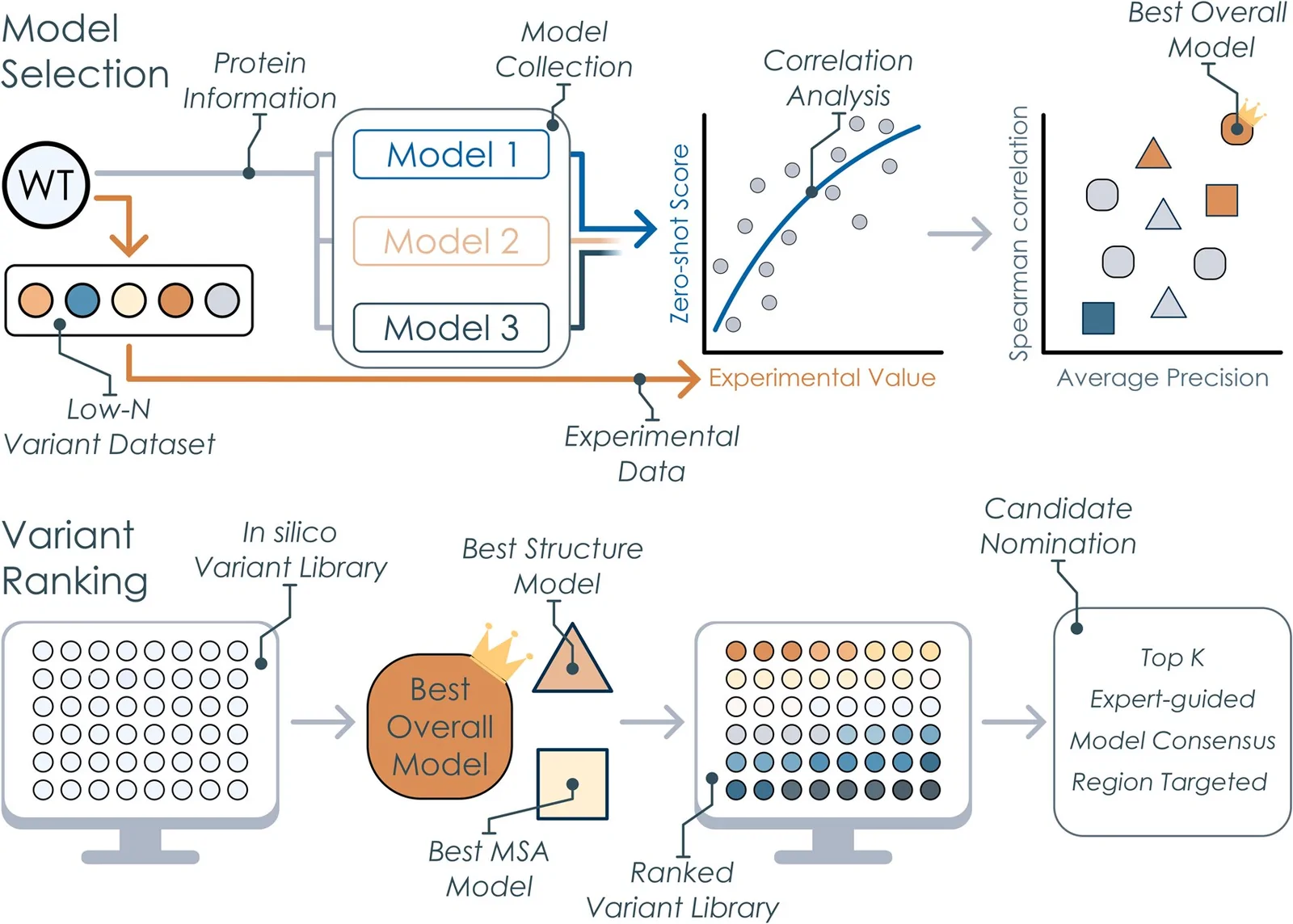
Overview of PRIZM workflow. The workflow consists of two phases: Model Selection and Variant Ranking. In the first phase, zero-shot predictors use the WT protein information to generate zero-shot scores for variants. These scores are compared to the experimental values in a low-N dataset of variants to identify the best-performing models. In the second phase, these models are applied to an *in silico* variant library to rank and nominate candidates for experimental characterization.

**Table 1:** Zero-shot models contained in the PRIZM collection.

Model^a^	**Scoring method** ^b^	Reference
***Sequence***		
** CARP** ** 600K, 38M, 76M, 640M**	Masked-marginal LLR	Yang *et al.* (2024)
**ESM-1b**	Masked-marginal LLR	Rives *et al.* (2021)
**ESM-1v**	Masked-marginal LLR	Meier *et al.* (2021)
**ESM-2** **8M, 35M, 150M, 650M, 3B**	Masked-marginal LLR	Lin *et al.* (2023)
**ProGen2 small, medium, base, large**	Autoregressive log-likelihood	Nijkamp *et al.* (2023)
**ProtGPT2**	Autoregressive log-likelihood	Ferruz *et al.* (2022)
** RITA** **small, medium, large, x-large**	Autoregressive log-likelihood	Hesslow *et al.* (2022)
**Tranception No Retrieval** **small, medium, large**	Autoregressive LLR	Notin *et al.* (2022a)
**UniRep**	Autoregressive log-likelihood	Alley *et al.* (2019)
***MSA***		
**eUniRep^c^**	Autoregressive log-likelihood	Biswas *et al.* (2021)
**EVE**	Evolutionary index	Frazer *et al.* (2021)
**GEMME**	Evolutionary conservation score	Laine *et al.* (2019)
**TranceptEVE** **small, medium, large**	Autoregressive LLR + EVE retrieval	Notin *et al.* (2022b)
**Tranception** **small, medium, large**	Autoregressive LLR + MSA retrieval	Notin *et al.* (2022a)
**MSA Transformer**	Masked-marginal LLR	Rao *et al.* (2021)
***Structure***		
**ESM-IF1**	Inverse-folding log-likelihood	Hsu *et al.* (2022b)
**MIF**	Masked-marginal LLR	Yang, *et al.* (2023)
**MIFST**	Masked-marginal LLR	Yang *et al.* (2023)
**MULAN** **small**	Masked-marginal LLR	Frolova *et al.* (2025)
**ProteinMPNN**	Inverse-folding log-likelihood	Dauparas *et al.* (2022)
**ProSST** 20, 128, 512, 1024, 2048, 4096	Masked-marginal LLR	Li *et al.* (2024a)
**ProtSSN** **20, 128, 512, 1024, 2048, 4096**	Masked-marginal LLR	Tan *et al.* (2025b)
**SaProt** **650M**	Masked-marginal LLR	Su *et al.* (2024)
***All***		
**RSALOR**	Conservation & solvent accessibility	Tsishyn *et al.* (2025)
**VenusREM** **20, 128, 512, 1024, 2048, 4096**	Masked-marginal LLR	Tan *et al.* (2025a)

a Rows under models specify model versions contained in PRIZM.

b LLR = log-likelihood ratio relative to the wild type values (WT); MSA = multiple sequence alignment; EVE = evolutionary model of variant effect.

c While eUniRep does not align related sequences, we still characterize it as an MSA-based model due to utilizing evolutionarily relevant information.

First, we validated the performance of PRIZM across 10 literature benchmark datasets with a diverse set of protein properties. For all datasets except one, we found the workflow to consistently differentiate between models with high or low correlation to the assayed properties using as few as 20 variants, although 50 variants were generally preferred for identifying the best-performing models.

We further evaluated the workflow in two additional protein systems: sucrose synthase (SuSy) thermostability and UDP-dependent glycosyltransferase (GT1) activity. In the first case study, we utilized an in-house pre-existing dataset of 68 variants from a semi-rational engineering campaign as the low-N dataset for model selection (Mejia-Otalvaro *et al.* 2026). Based on a combination of the three best models identified by PRIZM, we identified a variant with an apparent melting temperature increase (Δ*T*_m, app_) of ∼3.0°C. In the GT1 activity study, the low-N dataset consisted of only eight variants from a previous rational campaign (Lax *et al.* 2026). By using zero-shot predictions to guide variant prioritization, we identified a variant with ∼20% increase in relative activity, with an overall hit rate of ∼43% for identifying variants with enhanced relative activity compared to WT.

Overall, our study highlights how PRIZM facilitates easy and data-efficient engineering of proteins while requiring little computational setup. We believe PRIZM can guide non-experts in utilizing zero-shot predictions for candidate selection, as well as provide experts with an informed choice of foundation models for downstream modelling.

## 2 Methods

### 2.1 PRIZM workflow overview

The core of the PRIZM workflow comprises three main steps: query protein data pre-processing, the Model Selection phase, and the Variant Ranking phases (Fig. 1). We elaborate on each of the steps below.

#### 2.1.1 Protein data pre-processing

As a pre-processing step, we collect the protein information required as inputs for the pre-trained zero-shot models: the sequence, structure, and MSA. If no crystal structure without gaps is available, a structure is predicted by tools such as AlphaFold3 (Abramson *et al.* 2024) using the WT sequence. The WT sequence is also utilized to construct an MSA using the EVcouplings pipeline (Hopf *et al.* 2019), though a custom MSA can be employed if it has the correct format (A2M). Importantly, the resulting structure and MSA are used as zero-shot model inputs for all variants, not just WT. The pre-processing step produces two files: a low-N input dataset file with mutant specification, sequence, and experimental score, and a reference file with structure and MSA specifications.

#### 2.1.2 Model Selection—zero-shot processing

Following pre-processing, the first part of the Model Selection phase uses the input dataset to run a diverse collection of zero-shot models. The collection of models is based on the work done by Notin *et al.* (2023) in ProteinGym. The current version of PRIZM consists of 25 models in total—nine models using sequence as input, six MSA-based models, eight models using structural inputs, and two models using all three types of information (Table 1). PRIZM runs the models through a global wrapper module, where each model outputs a single scalar zero-shot score per variant, such as a masked-marginal log-likelihood ratio relative to WT or a conservation-based score (see Table 1 for details). As MSA training is a prerequisite for some zero-shot scorers, models that require significant pre-training on MSA data, such as EVE (Frazer *et al.* 2021) and evo-tuned UniRep (Alley *et al.* 2019, Biswas *et al.* 2021), are processed before others. Importantly, no experimental data are used for finetuning or influencing any of the models. Furthermore, PRIZM is not limited to only the models utilized in this study but can be extended to incorporate any newly released zero-shot model. For a thorough description of the individual models, we refer the reader to the original papers of the models and the description available at ProteinGym.

#### 2.1.3 Model Selection—model scoring

In the second part of the Model Selection phase, PRIZM determines the relationship between the zero-shot scores from each model and the actual experimental values. Absolute Spearman correlation is used to examine the ranking performance of each model across the full range of experimental values. Absolute rather than signed correlation is used, as an anti-correlated model is equally informative for variant ranking. Then, a user-defined threshold, such as the WT value, is used to binarize the experimental values and calculate the average precision, which specifically evaluates the model’s ability to classify high-performing variants above the threshold. While absolute Spearman correlation is already bounded in [0, 1], the average precision is min–max normalized with the random-baseline average precision, equal to the positive-class prevalence in the binarized dataset, as minimum and 1 as maximum. The performance metrics are added together, resulting in a single performance metric for each zero-shot model. The metric describes how well each model can explain the experimental property and is used for identifying the best and worst models for the given low-N dataset. For users who wish to restrict model selection to correctly signed correlated models or to substitute alternative performance metrics entirely, these options are available within the PRIZM workflow. The availability of alternative metrics within the workflow allows users to assess this directly for their specific system. Importantly, PRIZM not only outputs the highest- and lowest-performing models overall, but also the best and worst models using either sequence, MSA, protein structure, or all three information types. This allows the selection of multiple high-performing models based on different protein information, a strategy shown in this work to result in enhanced variants.

#### 2.1.4 Variant Ranking

In the second phase of PRIZM, the Variant Ranking phase, an *in silico* library is created by mutating the WT sequence. PRIZM allows specifying which regions to mutate, what residues to use, and how many substitutions to conduct, with the default setup being a full single-point mutagenesis of the WT protein. The library is written in the same format as the low-N input file and re-enters the scoring pipeline via a new reference file. The library is processed by the best models. These zero-shot scores are used to rank all variants in the library, using the original correlation analysis of the low-N dataset. The final ranked *in silico* library is returned as a sorted CSV-file that can be utilized as a tool for identifying new variants. Here, the nomination strategy can either be based on a single model score or an aggregate ranking from multiple models, using either a *greedy Top K* selection or employing a targeted approach where ranking and expert knowledge are combined. Detailed guidance on how to use PRIZM can be found in Supplementary Methods, available as supplementary data at *Bioinformatics Advances* online. Lastly, visualization utilities, e.g. correlation plots, model performance comparisons, and single-mutant landscapes, are provided as part of the analysis notebooks.

### 2.2 Validation of PRIZM on ProteinGym datasets

PRIZM is a model-selection framework rather than a supervised predictor trained on experimental data. The zero-shot models are applied directly, without fine-tuning or parameter optimization, and the role of the experimental low-N dataset is therefore not to train a new model but to identify which pre-trained predictor best captures the target property. Conventional train/test splitting is thus not directly applicable. Instead, to assess whether PRIZM can identify high-performing predictors from limited labelled data, we used a subsampling procedure in which low-N datasets of varying sizes were repeatedly drawn from full DMS datasets. These low-N subsets were used only for model selection, after which the selected models were evaluated on the corresponding full DMS datasets. This design therefore assesses the robustness of PRIZM as a model-selection framework, rather than model generalization in the supervised learning sense.

To obtain DMS benchmark datasets for the PRIZM validation, we selected 10 datasets from the ProteinGym dataset collection (Notin *et al.* 2023), a state-of-the-art collection of mutational datasets that have been used to validate and benchmark various engineering approaches. The selected datasets cover a diverse range of protein properties and measurement modalities, including protein aggregation (Seuma *et al.* 2022), receptor activity (Jones *et al.* 2020), thermostability (Nutschel *et al.* 2020), inhibitor resistance (Brenan *et al.* 2016), fluorescence (Ellis *et al.* 2024), receptor binding (Starr *et al.* 2020), peptide binding (Araya *et al.* 2012), and enzyme activity (Romero *et al.* 2015, Chiasson *et al.* 2020, Hobbs *et al.* 2022) (Table S1, available as supplementary data at *Bioinformatics Advances* online). The seven non-enzyme datasets were chosen to collectively span distinct property types, while three datasets measuring enzyme activity were included to ensure coverage of catalytic function, a primary target in protein engineering. The only exclusion criterion applied was that datasets had to contain single-point variants, excluding datasets consisting exclusively of multi-point mutations. It should be noted that we leveraged the corresponding MSA and structure files described in ProteinGym for PRIZM inputs. From each full DMS dataset, we generated low-N subsets by subsampling between 10 and 200 variants, repeating this subsampling procedure 100 times (Fig. S1, available as supplementary data at *Bioinformatics Advances* online). Both the full datasets and the corresponding low-N subsets were processed with PRIZM to identify high- and low-performing models. For each low-N dataset size, the models identified as best or worst by PRIZM were compared with their corresponding performance on the full dataset, and average performance across the 100 subsampling iterations was computed.

To compare the difference in performance of PRIZM between the best- and worst-performing models across the low-N dataset sizes, we calculated Cohen’s *d*, the standardized effect size:


$$d=\;\frac{\bar{B}-\bar{W}}{\sqrt{\frac{\sigma_{B}^{2}+\sigma_{w}^{2}}{2}}}$$

where $\bar{B}$, $\sigma_{B}$, $\bar{W}$, and $\sigma_{W}$ are the means and standard deviations of the performances of the best and worst models, respectively, across the 100 iterations (Cohen 1988). Following Cohen, we interpret a *d*-value of below 0.2 to be a small difference, between 0.2 and 0.5 to be a medium difference, and above 0.5 to be a large difference.

To assess the effect of mutation count on the PRIZM performance, we separated the protein aggregation (Seuma *et al.* 2022) and peptide binding (Araya *et al.* 2012) benchmark datasets into purely single or double mutant subsets. From these, we generated corresponding low-N datasets, processed them with PRIZM, and evaluated the best- and worst-performing models on the full datasets. This resulted in four comparisons: (i) performance on the full single-mutant dataset using models selected from single-mutant low-N data; (ii) performance on the full single-mutant dataset using models selected from double-mutant low-N data; (iii) performance on the full double-mutant dataset using models selected from single-mutant low-N data; and (iv) performance on the full double-mutant dataset using models selected from double-mutant low-N data. We again utilized Cohen’s *d* to quantify the difference between the best- and worst-performing models.

To compare PRIZM’s performance to the consensus approach of Hie *et al.* (2024), we replicated their strategy on the benchmark datasets in this study. Using ESM-1b and the five ESM-1v versions, we identified variants that were predicted to be better than WT for multiple of these models. As described by Hie *et al.*, we adjusted the consensus model count to limit how many variants were nominated, iteratively increasing the number of models until fewer than 40 variants were proposed. Furthermore, we implemented a similar consensus approach for the top-performing PRIZM models across the four protein information types: sequence, MSA, structure, and all. These two consensus approaches were compared to a simple *greedy top 10* approach leveraging the best-performing PRIZM model. For each approach, we calculated the hit rate, with the performance of the two PRIZM-based strategies being based on 100 iterations using a low-N dataset of 50 variants.

### 2.3 Generation of PRIZM inputs for case study proteins

The MSAs of GmSuSy and TOGT1_1 were created using EVcouplings (Hopf *et al.* 2019) with a bitscore cutoff of 0.5 for both. For GmSuSy, both a monomeric and a tetrameric structure were generated using AlphaFold3 (Abramson *et al.* 2024), while we only generated a monomeric structure of TOGT1_1. Only the monomeric structures were utilized as input for PRIZM. To obtain the GmSuSy low-N dataset, we subsampled all single mutants from the consensus mutagenesis campaign described in Mejia-Otalvaro *et al.* (2026). The TOGT1_1 low-N dataset contained all single-mutant substitutions described by Lax *et al.* (2026).

### 2.4 Nomination of variants for experimental validation in case studies

To nominate new GmSuSy variants, we ranked every single-point mutant using Tranception No Retrieval, MIFST, and MSA Transformer. These ranks were added together, resulting in a composite ranking of each variant. This was used to select the top five best variants, from which three had already been characterized in the original low-N dataset. We therefore selected the last two, L731E and F468I, for experimental characterization.

To nominate new TOGT1_1 variants, we constructed a full single-mutant landscape using the VenusREM scores, identifying six positions with high predicted mutability: 53, 72, 208, 239, 389, and 401. At each position, we examined the TOGT1_1 structure to identify any steric clashes caused by the mutations. For all positions except 53, the highest-ranked mutation was chosen as no steric clashes were predicted by expert analysis. However, the best variant at 53, N53Y, showed potential for structural conflicts, and we therefore chose the second variant. Lastly, we selected the third-ranked variant at position 401, G401F, as (i) the site appeared able to accommodate a bulkier residue, and (ii) the Gly to Phe substitution introduced a substantial local change, making it an interesting mutation to test. Our final nomination of new TOGT1_1 variants for experimental characterization was N53A, N72S, D208L, W239I, E389A, G401I, and G401F.

### 2.5 Experimental validation

The experimental assays and materials used in the two case studies follow the original publications by Mejia-Otalvaro *et al.* (2026) and Lax *et al.* (2026). A summary of the experimental procedures is provided in Supplementary Methods, available as supplementary data at *Bioinformatics Advances* online.

### 2.6 Computational setup

All computations were performed on a Microsoft Azure Standard NC6s v3 virtual machine (6 vCPUs, 112 GiB RAM, NVIDIA Tesla V100 16 GB GPU, Ubuntu 24.04 LTS). PRIZM is implemented in Python and requires a Linux environment with CUDA-enabled GPU support; setup and usage instructions are provided in the GitHub repository: https://github.com/daha-la/PRIZM. Each model is associated with a designated conda environment in the PRIZM codebase, isolating models with conflicting dependencies while allowing compatible ones to share a common environment; a global submission script activates the appropriate environment per model and manages model ordering and execution mode (sequential or parallel), allowing the panel to be executed end-to-end from a single command. Most models utilize GPU acceleration, which is the primary computational bottleneck, while a subset of models run on CPU. Runtimes vary substantially across models, with the fastest models scoring a full single-mutant library within minutes and slower models requiring several hours. The most time-intensive step is the “evotuning” of UniRep, which can require approximately one day depending on MSA depth. Trained MSA-conditioned models (EVE and evo-tuned UniRep) are cached and reused across runs sharing the same MSA, including the *in silico* library generated in the Variant Ranking phase, which substantially reduces total runtime when scoring large libraries. Overall, the computational requirements are modest by modern standards and accessible on standard cloud computing infrastructure.

## 3 Results

To leverage low-N datasets while avoiding the pitfalls of supervised training based on limited data, we investigated whether it is possible to use a low-N dataset of experimental labels only to identify the most suitable zero-shot models for a given engineering task. To this end, we assembled a simple workflow PRIZM consisting of two phases: the Model Selection phase, where the best zero-shot model is identified, and the Variant Ranking phase, where the best model is used to predict and rank an *in silico* library (Fig. 1). In what follows, we first evaluated the performance of such an approach retrospectively based on a selection of deep mutational scanning (DMS) datasets from the ProteinGym dataset collection. We then tested it prospectively on two different protein systems: a SuSy and a GT1.

### 3.1 Validation of PRIZM on ProteinGym datasets

To examine the performance of PRIZM, we first validated the workflow on benchmark DMS datasets (Fig. 2). We compared the performance of the models selected as “best” and “worst” by PRIZM, meaning those with the highest and lowest combined Spearman correlation and average precision, respectively (Fig. 2A, Figs S2–S4, available as supplementary data at *Bioinformatics Advances* online). Except for the inhibitor resistance of Mitogen-activated protein kinase 1 (MAPK1) (Brenan *et al.* 2016), we observed a Cohen’s *d* > 0.5 for the separation between best and worst models using datasets containing as few as 20 variants [30 for enzyme activity of epoxide reductase (Chiasson *et al.* 2020)]. As *d* > 0.5 denotes a large difference, this suggests that ∼20 variants are sufficient for PRIZM to reliably distinguish between high- and low-performing models. Datasets below this threshold may yield unreliable estimates of Spearman correlation and average precision, as both metrics exhibit substantially higher variance when estimated from fewer than ∼20 variants (Figs S5 and S6, available as supplementary data at *Bioinformatics Advances* online). Moreover, using ∼50 variants generally led to models with performance similar to the zero-shot upper limit, meaning the model with the overall best performance on the entire dataset, and further increases in the labelled dataset size used for model selection yielded only minor improvements. Model ranking was found to be robust across a range of binarization thresholds from the 20th to the 80th percentile of the experimental distribution, for both the full dataset and subsamples of 50 variants (Fig. S7, available as supplementary data at *Bioinformatics Advances* online).

**Figure 2:**
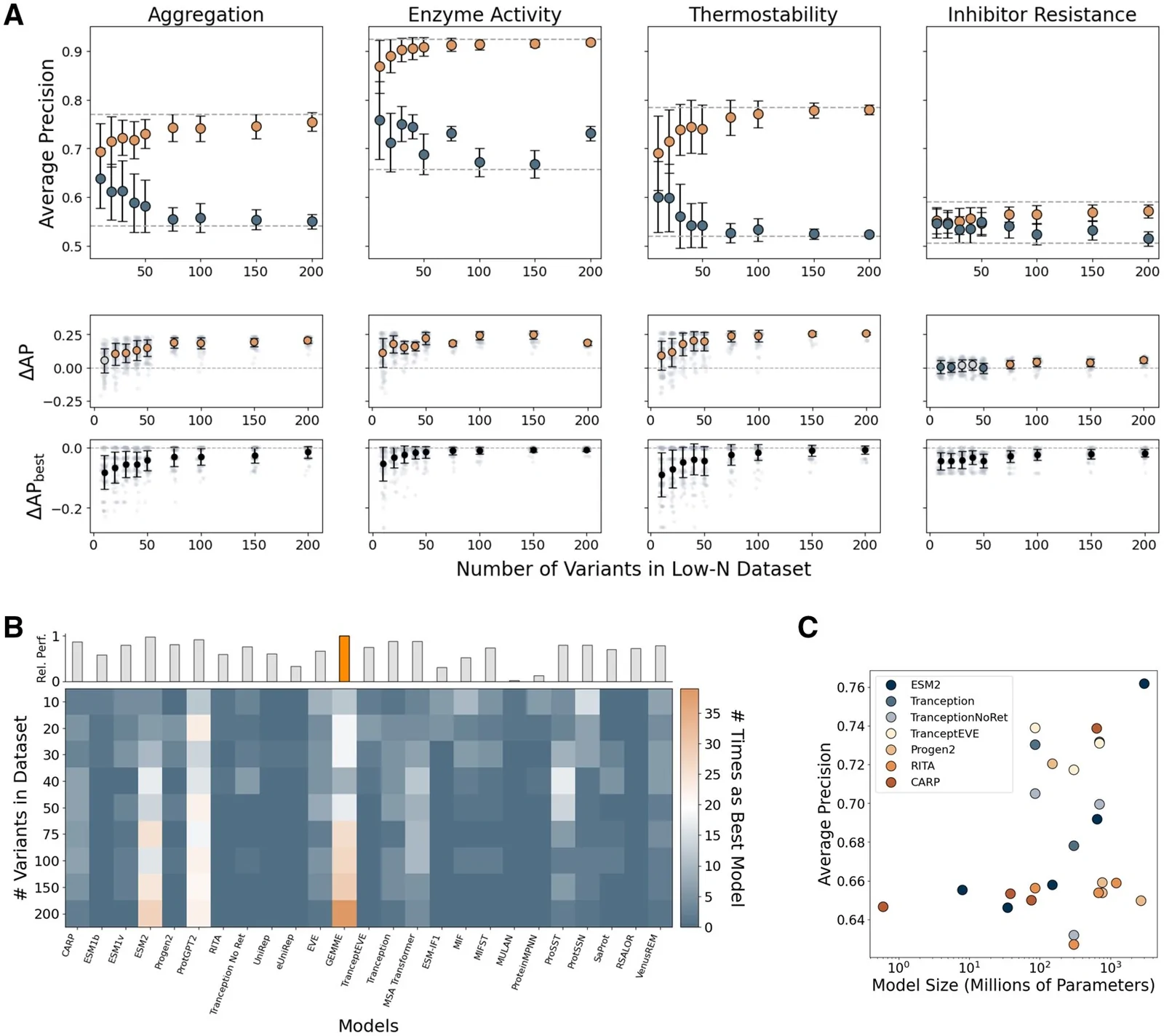
Validation of PRIZM workflow on ProteinGym datasets. (A) Performance of zero-shot models selected by PRIZM for predicting full DMS datasets as a function of the number of variants in the low-N dataset [aggregation data from Seuma *et al.* (2022), enzyme activity from Hobbs *et al.* (2022), thermostability from Nutschel *et al.* (2020), inhibitor resistance from Brenan *et al.* (2016)]. The top row shows average precision (AP) for the best (orange) and worst (blue) models as identified by PRIZM, with dotted lines indicating the zero-shot upper and lower limit on the whole dataset. The middle row shows ΔAP between the best and worst models, colored by Cohen’s *d* (blue: *d* < 0.2, grey: *d* < 0.5, orange: *d* > 0.5). The bottom row shows ΔAP between each selected model and zero-shot upper limit on the entire dataset (ΔAP_best_). Error bars represent the standard deviation from 100 PRIZM iterations. (B) Frequency of each model being selected as the best by PRIZM for different low-N dataset sizes for the aggregation DMS dataset from Seuma et al. (2022) across 100 iterations. The top inset shows each model’s relative performance by comparing its average precision with that of the overall best model (orange) on the full dataset. (C) Average model precision as a function of model size for the aggregation DMS dataset from Seuma *et al.* (2022).

**Figure 3:**
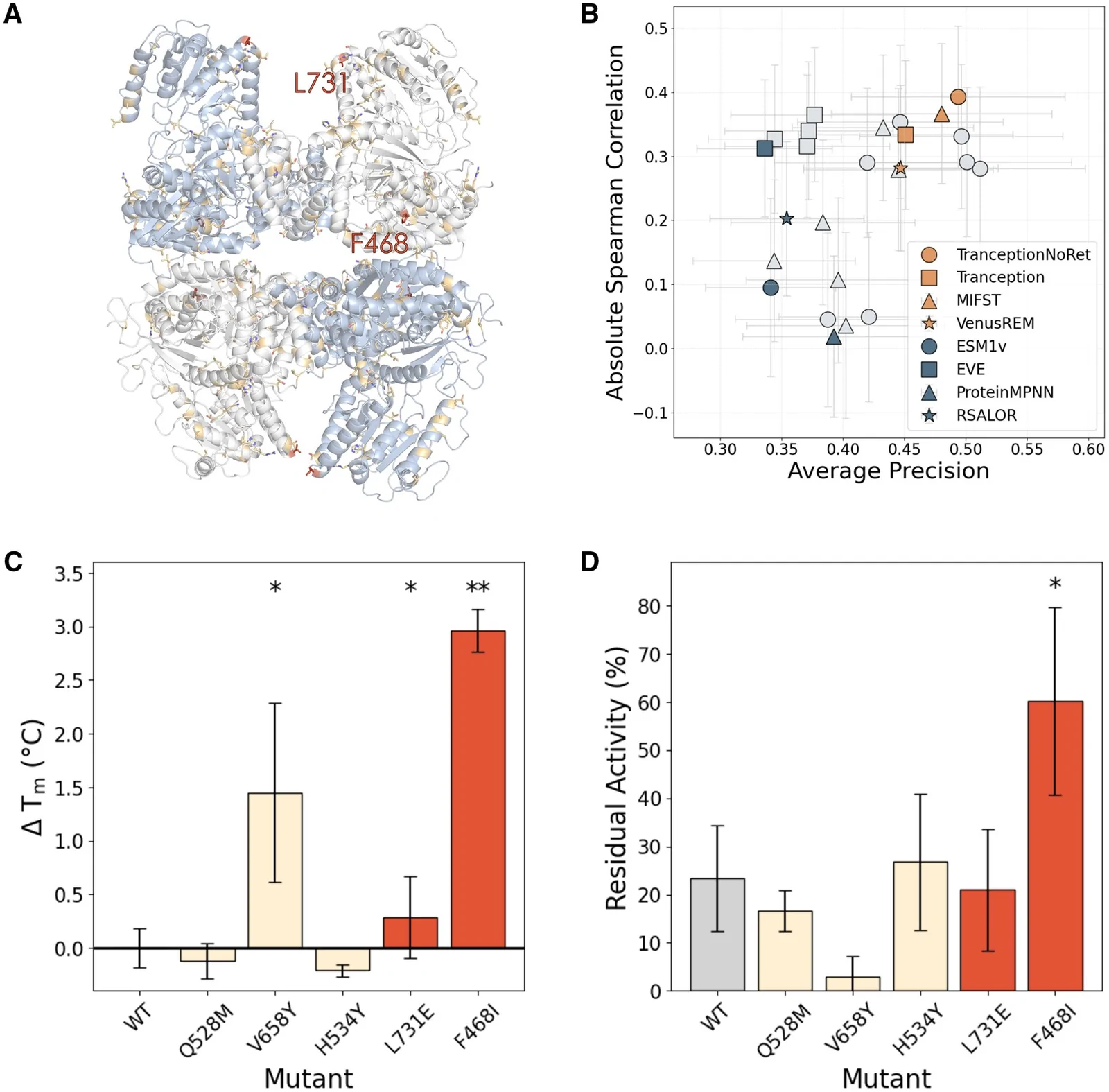
PRIZM engineering of GmSuSy. (A) A predicted structure of the GmSuSy tetramer using AlphaFold3. Positions mutated in the original consensus mutagenesis campaign by Mejia-Otalvaro *et al.* (2026) are shown in orange, while the positions mutated in this study are shown in red. (B) Spearman correlation and average precision of the PRIZM zero-shot models for predicting GmSuSy *T*_m, app_. Circle = sequence-based model, square = MSA-based model, triangle = structure-based model, star = all three. Blue and orange marker colours denote the lowest and highest performing models for each model category, respectively, as identified by PRIZM. Error bars indicate the standard deviation evaluated from bootstrapping the initial dataset (*N* = 1000). (C, D) The experimental Δ*T*_m,app_ (C) and residual activity after 5 min incubation at 60°C (D) of WT GmSuSy and the five expressed variants predicted to be better than WT by Tranception No Retrieval, MSA Transformer, and MIFST, sorted by the combined rank. Grey = WT, beige = variants from previous campaign, red = new variants identified by PRIZM. Asterisks denote relative activities above WT (one-sided Welch test with *α* = 0.05 or *α* = 0.01, indicated by one or two asterisks, respectively), while the error bars indicate error propagated replicate standard deviation (*N* = 3).

**Figure 4:**
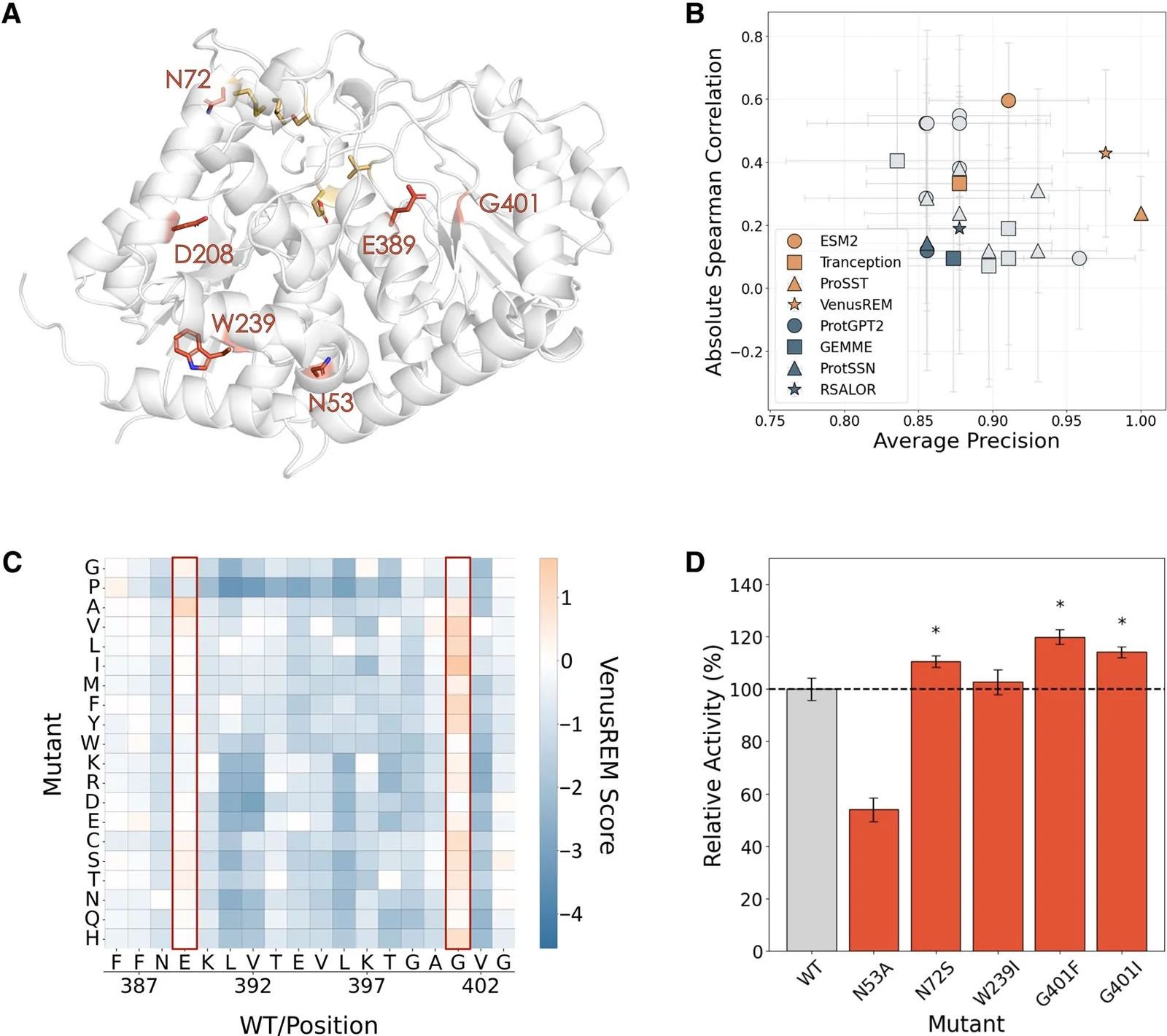
PRIZM engineering of TOGT1_1. (A) A predicted structure of TOGT1_1 using AlphaFold3. Positions mutated in the original rational design dataset from Lax *et al.* (2026) are shown in beige, while the positions mutated in this study are shown in red. (B) Spearman correlation and average precision of the PRIZM zero-shot models for predicting TOGT1_1 relative activity. Circle = sequence-based model, square = MSA-based model, triangle = structure-based model, star = all three. Blue and orange denote the worst and best models for each model category, respectively, as identified by PRIZM. Error bars indicate the standard deviation using a bootstrapping approach (*N* = 1000). (C) Representative section of TOGT1_1 *in silico* landscape of VenusREM scores. Orange denotes variants predicted to be better than WT, while blue denotes variants predicted to be worse. Red squares mark E389 and G401; selected for experimental characterization due to high proposed mutability. (D) Relative activities of TOGT1_1 variants selected using the TOGT1_1 VenusREM landscape. Asterisks denote relative activities above WT (one-sided Welch test with *α* = 0.05), while the error bars indicate replicate standard deviation (*N* = 3).

We further examined how often the models with the highest overall performance of the full DMS datasets were chosen by PRIZM as a function of the low-N dataset size (Figure 2B, Figs S8–S16). Here, we found that PRIZM generally converged towards high-performing models using low-N datasets of 50 variants or more. For seven out of the ten datasets, our workflow successfully identified the model with the highest overall performance, with the required number of variants ranging from 50 to 200. The identity of the top-performing model also varied across datasets, underscoring that no single model consistently dominated performance.

Regarding model scaling, some studies have shown an increase in performance by increasing the model size (Bhatnagar *et al.* 2025, Candido *et al.* 2026), while others have reported that scaling benefits are task-dependent, with little gain on most property prediction tasks and reduced accuracy beyond a certain model size for zero-shot fitness prediction (Notin 2025). Our results are consistent with the latter reports, as we observed that increasing the number of model parameters did not necessarily lead to improved performance (Fig. 2C, Fig. S17, available as supplementary data at *Bioinformatics Advances* online). As model size is not a reliable proxy for performance, model choice cannot be reduced to selecting the largest available model. In summary, our analysis highlights not only the general robustness of PRIZM for selecting high-performing models but also the importance of the Model Selection Phase as a whole.

Next, we assessed the impact of mutation count per variant by evaluating PRIZM on datasets restricted to single or double mutants (Figs. S18 and S19, available as supplementary data at *Bioinformatics Advances* online). We found a general decrease in performance of the zero-shot models when predicting the effects of double mutants, which could potentially be caused by the models’ poor understanding of epistatic effects. PRIZM’s ability to distinguish between high- and low-performance models also appears to weaken in the case of learning from double mutants for datasets with fewer variants. This is consistent with recent studies observing how zero-shot-based methodologies struggle to predict the effect of epistasis (Kolchina *et al.* 2026, Visani *et al.* 2026). It is therefore still an open question how to properly use zero-shot models to capture epistasis.

Lastly, to compare the performance of PRIZM to a previous zero-shot prediction workflow, we compared PRIZM with the approach described by Hie *et al.* (2024). First, we applied their approach to the benchmark datasets described in this study. Secondly, we assessed the hit rate of PRIZM of the top 10 variants ranked by the top-performing model. As a last step, we adapted the consensus approach of Hie *et al.*, using the best models of each protein information type. PRIZM outperformed the approach of Hie *et al.* (2024) across six of the 10 benchmark datasets (Fig. S20, available as supplementary data at *Bioinformatics Advances* online). Furthermore, a key limitation of the Hie *et al.* approach is that it can only propose mutations that are predicted to outperform WT by multiple zero-shot models simultaneously. If no such variants exist for a given protein, the method cannot nominate any candidates. On the contrary, PRIZM always provides a ranked *in silico* library of any chosen model. This gives researchers full flexibility in how they prioritize mutations, whether by employing a *greedy Top K* approach, combining multiple model rankings, or incorporating expert domain knowledge.

In summary, our validation highlights the versatility and generalizability of PRIZM for model selection and variant prioritization. The approach extends beyond enzyme activity to protein properties such as thermostability, receptor activity, and fluorescence, reliably distinguishing high- and low-performing models with as few as 20 variants, and as few as 50 variants are usually enough to reach the upper limit on the performance, based on the retrospective analysis of published datasets. The method can handle double mutants as well, although a lower average precision is achieved when predicting the effects of double-point mutations. Overall, PRIZM provides a scalable, property-agnostic strategy for navigating the space of zero-shot models while benefiting from available small experimental datasets.

To demonstrate how PRIZM can help guide protein engineering campaigns in real applications, we next engineered the thermostability of SuSy and catalytic activity of a GT1.

### 3.2 Applying PRIZM to improve the thermostability of SuSy using an existing dataset

As mentioned previously, state-of-the-art MLDE approaches often emphasize the creation of new, unbiased datasets (Jiang *et al.* 2025). Here, we evaluated PRIZM’s ability to utilize existing datasets, thereby minimizing the requirement for new experimental characterization.

As a case study, we investigated the thermostability of SuSy from Glycine max (GmSuSy). SuSy is able to catalyze the reversible transfer of glucose from sucrose to nucleotide diphosphates (typically UDP), forming UDP-glucose and fructose. Owing to this activity, SuSy enzymes are frequently employed in UDP-glucose regeneration systems for GT1-coupled reactions (Mejia-Otalvaro *et al.* 2025).

We have previously shown how the engineering of GmSuSy thermostability can be guided using tools such as ancestral sequence reconstruction and consensus mutagenesis (Mejia-Otalvaro *et al.* 2026). Based on the latter, we obtained a dataset consisting of 68 single mutants distributed across the full protein sequence and their melting temperature (*T*_m,app_) (Fig. 3A). This dataset was used as input for PRIZM to identify the most suitable zero-shot model for predicting *T*_m,app_ (Fig. 3B, Figs S21 and S22, available as supplementary data at *Bioinformatics Advances* online). Based on our PRIZM validation, a low-N dataset of 68 variants should generally yield high-performing models using PRIZM. Among the top-performing models, two demonstrated comparable performance: Tranception No Retrieval and MIFST, which rely primarily on sequence and structural information, respectively. We selected both models along with MSA Transformer, the second-best-performing MSA-based model. We did not choose the best MSA-based model, Tranception, to avoid potential redundancy with two Tranception-based models.

We then processed an *in silico* library of all possible single-point variants using the three selected models and identified the variants predicted to outperform WT across all models (Fig. S23, available as supplementary data at *Bioinformatics Advances* online). Notably, the resulting list included several variants that were already present in the original rationally designed dataset, showing a convergence between expert-driven rational mutagenesis and zero-shot predictions, even though the algorithm is naïve to said expert-driven mutagenesis. When ranking variants by their combined performance across the three models, these previously characterized mutants occupied three of the top five positions, with the standout variant V658Y having a Δ*T*_m,app_ of ∼1.7°C (Fig. 3C, Table S2, available as supplementary data at *Bioinformatics Advances* online). We further characterized the remaining two variants, absent from the original dataset, L731E and F468I, which both had higher Δ*T*_m,app_ than WT (∼0.3°C and ∼3.0°C, respectively). Overall, while moderate in the absolute numbers, our PRIZM analysis identified two new variants with higher *T*_m,app_ than WT. Furthermore, the best variant, F468I, outperformed all variants in the original dataset (Fig. 3C, Table S2, available as supplementary data at *Bioinformatics Advances* online). This increase in melting temperature was accompanied by kinetic stability towards elevated temperature, as supported by residual activity assays at 60°C, where F468I retained over 60% activity, compared to ∼23% for WT (Fig. 3D). This concordance is not guaranteed, as stability and activity can decouple, with gains in one occurring at the expense of the other (Escobedo *et al.* 2025, Raechell *et al.* 2026).

### 3.3 Applying PRIZM to engineer a GT1 using fewer than 10 variants as initial dataset

To assess the robustness of PRIZM’s Model Selection Phase, we evaluated its performance in an extremely low-N setting. In our prior work, we conducted an engineering campaign of the GT1 from Nicotiana attenuata (TOGT1_1) (Lax *et al.* 2026). GT1s catalyze the transfer of sugar moieties to a diverse set of acceptor molecules, offering a biocatalytic solution to the synthesis of industrially relevant glycosylated compounds (Gharabli *et al.* 2023, Sirirungruang *et al.* 2023). We focused on niclosamide, an essential small molecule therapeutic that suffers from low water solubility, which limits its therapeutic use beyond tapeworm treatment. We previously identified TOGT1_1 as a niclosamide-active GT1 (Harding-Larsen *et al.* 2024b, Lax *et al.* 2026) and performed a small rational design campaign of eight single active site mutants to improve the catalytic efficiency, with the best mutant having 109% relative activity compared to WT.

We used this dataset of eight single mutants as input to PRIZM (Fig. 4A), through which PRIZM determined VenusREM to be the top-performing model (Fig. 4B, Figs S24 and S25, available as supplementary data at *Bioinformatics Advances* online). This model integrates all three protein information types: sequence, structure, and MSA.

Similar to the previous PRIZM engineering campaign, we used VenusREM to process an *in silico* library of all possible single mutants. Importantly, the small size of the initial low-N dataset decreases the likelihood that we have identified a top-performing zero-shot model. Consequently, rather than fully trusting the model output and only selecting top-ranked substitutions, we incorporated expert curation. First, we analyzed the VenusREM mutational landscape to guide variant selection. Here, we identified positions with high apparent mutability, defined as those where VenusREM predicted the majority of substitutions to result in enhanced performance (Fig. 4C, Fig. S26, available as supplementary data at *Bioinformatics Advances* online). VenusREM predicted the positions of 53, 72, 208, 239, 389, and 401 to have high predicted mutability, and seven single mutants were selected for further characterization based on a combination of zero-shot ranking, chemical knowledge, and protein structure examination (Fig. 4D, Table S3, available as supplementary data at *Bioinformatics Advances* online), the latter being common practice for eliminating computationally proposed substitutions that have clear structural problems (Wijma *et al.* 2014). For N72, D208, W239, and E389, we chose the highest-ranked variant, while we chose an Ala substitution instead of Tyr for N53 due to apparent backbone restrictions. For G401, we selected both the highest-ranked variant and the third-highest-ranked variant, due to the latter representing a drastic biochemical change from Gly to Phe. We were unable to express two of these variants, but of the expressed mutants, we identified N72S, G401F, and G401I, with relative activities of 110.5%, 119.9%, and 114.1% compared to the WT, respectively. This corresponded to a hit rate of ∼43% for variants better than WT (three out of the seven nominated). While the observed improvements are small in magnitude, the best-performing variants, G401F and G401I, perform comparably to the best variants from the original engineering campaign. Furthermore, G401F and G401I are situated in a coil-rich region distant from the active site, which would likely be overlooked in a rational design campaign. This is consistent with previous reports that substitutions at distal Gly positions are among the strongest modulators of ligand binding (Faure *et al.* 2024, Weng *et al.* 2024). Interestingly, the best variant, G401F, was ranked as the third best G401 substitution (11th best overall) by VenusREM. This underscores the strength of combining zero-shot predictors and expert evaluation for exploring the protein sequence space.

## 4 Discussion

This study describes PRIZM, a two-phase approach for leveraging pre-trained zero-shot models to predict the effects of protein variants, offering a robust and accessible protein engineering workflow for non-experts. By identifying the zero-shot models that best describe a target protein property, PRIZM combines the simplicity of zero-shot modelling with the task-specific insight typically associated with supervised learning. In doing so, it addresses two major challenges: the difficulty of supervised ML in low-N settings and the lack of guidance in standard zero-shot modelling, all without requiring model training, dataset construction, or specialized ML knowledge.

Importantly, our work does not claim that PRIZM is superior to supervised learning. Instead, PRIZM represents an orthogonal strategy to supervised approaches as it focuses on the selection of a suitable pre-trained model rather than its integration in downstream training on low-N data. Often, such a selection in the latter case is done either arbitrary or based on a global validation across multiple benchmark datasets. Recent research increasingly points out that decisions based on broad benchmarks might be suboptimal in particular case studies (Woolley *et al.* 2026). Hence, PRIZM can also be incorporated in such workflows so as to enable researchers to select a model and generate zero-shot scores or protein embeddings that are property- and protein-specific.

In practice, PRIZM is particularly useful in three scenarios: when a small mutational dataset from a previous engineering campaign is already available, when experimental budget constraints limit the feasibility of multiple iterative design rounds, and when researchers aim to leverage zero-shot predictors without extensive ML expertise. Here, our benchmarking suggests that PRIZM can generally distinguish high- and low-performing models using ∼20 variants, while results below this regime should be interpreted with caution. Furthermore, when running PRIZM, it is important to first verify that the best model shows performance above a random baseline. As shown in Figs S5 and S6, available as supplementary data at *Bioinformatics Advances* online, in the case of a low-N dataset of 15 variants with 30% positive labels, having a Spearman correlation below 0.27 or an average precision below 0.58 for the best model would generally indicate that none of the tested foundation models capture the patterns in the collected data. As PRIZM can never be better than the best model, alternative strategies should be used instead, such as physics-based computational modelling or directed evolution. Nonetheless, the TOGT1_1 case study indicates that even in very low-N settings, PRIZM may still provide useful guidance when combined with expert curation of the ranked candidates.

We validated PRIZM across 10 benchmark DMS datasets, finding that the models ranked as best by PRIZM consistently outperformed the lowest-ranked ones using as few as 20 variants; the best models chosen using only 50 variants ranked among the top performers from the full DMS datasets. We further conducted two case studies targeting the thermostability of GmSuSy and the activity of TOGT1_1, illustrating the application of PRIZM to distinct engineering tasks across different target proteins and properties in contexts constrained by low-throughput experimental assays. Our validation and case studies also highlight the framework’s ability to leverage data from previous engineering campaigns, thereby opening new opportunities for researchers to repurpose existing experimental datasets for model-driven protein design.

Beyond serving as a tool for zero-shot-based protein engineering, PRIZM can also act as an initial step within a larger design pipeline, potentially mitigating some of the limitations of PRIZM. In particular, the quality of variant predictions is inherently constrained by the accuracy of the best-performing zero-shot model. As highlighted when processing the inhibitor resistance dataset of MAPK1 (Brenan *et al.* 2016), PRIZM may struggle with properties for which no pre-trained model adequately captures the relevant mutational effects. In the case of MAPK1, the inhibitor resistance was found to be uncorrelated with protein function. Zero-shot models learn from evolutionary sequence variation and therefore capture functional constraint well but carry no signal for resistance to a synthetic inhibitor that was absent from evolutionary history. As such, zero-shot predictions are expected to struggle with properties governed by protein–drug interactions, regulatory evasion, or other context-specific mechanisms that leave no imprint on sequence evolution. Even when PRIZM can capture a specific property, it does not explain the overall effect of the mutation or the resolution of the prediction due to operating purely on the basis of correlation with zero-shot scores. Additionally, the ranking of double mutant libraries revealed potential challenges with modelling epistatic interactions. These limitations could be addressed by integrating PRIZM into a supervised framework that leverages the best zero-shot model. As shown in the ProteinGym benchmark study, the best supervised approaches generally outperform the purely zero-shot-based ones (Notin *et al.* 2023). A PRIZM-based approach could entail substituting the ESM2 embeddings of the EVOLVEpro workflow with those from the top-performing embedding model (Jiang *et al.* 2025), or augmenting the zero-shot score with a one-hot encoding as described by Hsu *et al.* (2022a). However, a thorough comparison of zero-shot and supervised approaches is out of the scope of this study.

Another key challenge when using PRIZM lies in selecting variant candidates for further characterization. Relying solely on a *greedy Top K* selection strategy limits the sequence exploration, while incorporating expert knowledge risks diminishing the contribution of the latent biological patterns captured by large foundation models. In our current study, we chose to introduce the additional curation step when the dataset size was below 50 data points, the estimate obtained based on retrospective evaluation of PRIZM on ProteinGym datasets. While such a decision proved working in the case studies we considered, their limited number does not allow us to draw any general conclusions. Hence, the question of whether engineering campaigns should mainly follow the zero-shot scores or utilize manual expert curation remains relevant and largely open. A potential orthogonal solution could involve employing Bayesian optimization, as proposed by Hie and Yang (2022), where the model uncertainty guides the selection of new variants.

Collectively, these considerations underscore PRIZM’s potential to serve as a foundation for bridging zero-shot modelling and data-driven protein design. We envision PRIZM as a resource to inform and accelerate future protein engineering efforts for experts and non-experts alike, whether employed as an independent predictive tool or integrated within larger design pipelines.

## Supplementary Material

vbag299_Supplementary_Data

## Data Availability

All code and datasets used to produce the results and figures in this article are publicly available via Zenodo (PRIZM v1.1.2; DOI: 10.5281/zenodo.23035983) and the PRIZM GitHub repository (release v1.1.2; https://github.com/daha-la/PRIZM). The GitHub repository also includes documentation and installation/running instructions. The archived Zenodo release contains the complete codebase, analysis scripts used to reproduce the figures, and the datasets underlying this study.

## References

[vbag299-B1] Abramson J , Adler J, Dunger J et al Accurate structure prediction of biomolecular interactions with AlphaFold 3. Nature 2024;630:493–500. 10.1038/S41586-024-07487-W PMC11168924 38718835

[vbag299-B2] Alley EC , Khimulya G, Biswas S et al Unified rational protein engineering with sequence-based deep representation learning. Nat Methods 2019;16:1315–22. 10.1038/S41592-019-0598-1 PMC7067682 31636460

[vbag299-B3] Araya CL , Fowler DM, Chen W et al A fundamental protein property, thermodynamic stability, revealed solely from large-scale measurements of protein function. Proc Natl Acad Sci U S A 2012;109:16858–63. 10.1073/PNAS.1209751109 PMC3479514 23035249

[vbag299-B4] Arnold FH. Directed evolution: bringing new chemistry to life. Angew Chem Int Ed Engl 2018;57:4143–8. 10.1002/ANIE.201708408 PMC5901037 29064156

[vbag299-B5] Bhatnagar A , Jain S, Beazer J et al Scaling unlocks broader generation and deeper functional understanding of proteins. In: *Advances in Neural Information Processing Systems 38 Main Conference (NeurIPS 2025)*. 10.52202/085713-1536

[vbag299-B6] Biswas S , Khimulya G, Alley EC et al Low-N protein engineering with data-efficient deep learning. Nat Methods 2021;18:389–96. 10.1038/S41592-021-01100-Y 33828272

[vbag299-B7] Braun M , Gruber CC, Krassnigg A et al Accelerating biocatalysis discovery with machine learning: a paradigm shift in enzyme engineering, discovery, and design. ACS Catal 2023;13:14454–69. 10.1021/ACSCATAL.3C03417 PMC10629211 37942268

[vbag299-B8] Brenan L , Andreev A, Cohen O et al Phenotypic characterization of a comprehensive set of MAPK1/ERK2 missense mutants. Cell Rep 2016;17:1171–83. 10.1016/J.CELREP.2016.09.061 PMC5120861 27760319

[vbag299-B9] Candido S , Hayes T, Derry A et al Language modeling materializes a world model of protein biology. bioRxiv, 10.64898/2026.06.03.729735, 4 June 2026, preprint: not peer reviewed.

[vbag299-B10] Chiasson MA , Rollins NJ, Stephany JJ et al Multiplexed measurement of variant abundance and activity reveals VKOR topology, active site and human variant impact. Elife 2020;9:1–25. 10.7554/ELIFE.58026 PMC7462613 32870157

[vbag299-B11] Cohen J. Statistical Power Analysis for the Behavioral Sciences. 2nd edn. New York: Lawrence Erlbaum Associates, 1988.

[vbag299-B12] Dauparas J , Anishchenko I, Bennett N et al Robust deep learning-based protein sequence design using ProteinMPNN. Science 2022;378:49–56. 10.1126/SCIENCE.ADD2187 PMC9997061 36108050

[vbag299-B13] Ellis HJ , Chan M, Selvam B et al Deep mutagenesis of a transporter for uptake of a non-native substrate identifies conformationally dynamic regions. bioRxiv, 10.1101/2021.04.19.440442, 4 May 2024, preprint peer reviewed by *eLife* eeb.embo.org/doi/10.1101/2021.04.19.440442

[vbag299-B14] Escobedo A , Voigt G, Faure AJ et al Genetics, energetics, and allostery in proteins with randomized cores and surfaces. Science 2025;389:eadq3948. 10.1126/SCIENCE.ADQ3948 40705891

[vbag299-B15] Faure AJ , Martí-Aranda A, Hidalgo-Carcedo C et al The genetic architecture of protein stability. Nature 2024;634:995–1003. 10.1038/S41586-024-07966-0 PMC11499273 39322666

[vbag299-B16] Ferruz N , Schmidt S, Höcker B. ProtGPT2 is a deep unsupervised language model for protein design. Nat Commun 2022;13:4348. 10.1038/S41467-022-32007-7 PMC9329459 35896542

[vbag299-B17] Frazer J , Notin P, Dias M et al Disease variant prediction with deep generative models of evolutionary data. Nature 2021;599:91–5. 10.1038/s41586-021-04043-8 34707284

[vbag299-B18] Frolova D , Pak M, Litvin A et al MULAN: multimodal protein language model for sequence and structure encoding. Bioinform Adv 2025;5:vbaf117. 10.1093/BIOADV/VBAF117 PMC12452268 40989496

[vbag299-B19] Gharabli H , Della Gala V, Welner DH et al The function of UDP-glycosyltransferases in plants and their possible use in crop protection. Biotechnol Adv 2023;67:108182. 10.1016/J.BIOTECHADV.2023.108182 37268151

[vbag299-B20] Gurev S , Youssef N, Jain N et al Evaluating variant effect prediction across viruses. bioRxiv, 10.1101/2025.08.04.668549, 12 January 2026.

[vbag299-B21] Harding-Larsen D , Funk J, Madsen NG et al Protein representations: encoding biological information for machine learning in biocatalysis. Biotechnol Adv 2024a;77:108459. 10.1016/J.BIOTECHADV.2024.108459 39366493

[vbag299-B22] Harding-Larsen D , Madsen CD, Teze D et al GASP: a pan-specific predictor of family 1 glycosyltransferase acceptor specificity enabled by a pipeline for substrate feature generation and large-scale experimental screening. ACS Omega 2024b;9:27278–88. 10.1021/ACSOMEGA.4C01583 PMC11209901 38947828

[vbag299-B23] Hesslow D , Zanichelli NO, Notin P et al RITA: a study on scaling up generative protein sequence models. arXiv, https://arxiv.org/pdf/2205.05789, 11 May 2022, preprint: not peer reviewed (23 June 2025, date last accessed).

[vbag299-B24] Hie BL , Shanker VR, Xu D et al Efficient evolution of human antibodies from general protein language models. Nat Biotechnol 2024;42:275–83. 10.1038/S41587-023-01763-2 PMC10869273 37095349

[vbag299-B25] Hie BL , Yang KK. Adaptive machine learning for protein engineering. Curr Opin Struct Biol 2022;72:145–52. 10.1016/J.SBI.2021.11.002 34896756

[vbag299-B26] Hobbs HT , Shah NH, Shoemaker SR et al Saturation mutagenesis of a predicted ancestral Syk-family kinase. Protein Sci 2022;31:e4411. 10.1002/PRO.4411 PMC9601881 36173161

[vbag299-B27] Høie MH , Cagiada M, Beck Frederiksen AH et al Predicting and interpreting large-scale mutagenesis data using analyses of protein stability and conservation. Cell Rep 2022;38:110207. 10.1016/j.celrep.2021.110207 35021073

[vbag299-B28] Hopf TA , Green AG, Schubert B et al The EVcouplings Python framework for coevolutionary sequence analysis. Bioinformatics 2019;35:1582–4. 10.1093/BIOINFORMATICS/BTY862 PMC6499242 30304492

[vbag299-B29] Hsu C , Nisonoff H, Fannjiang C et al Learning protein fitness models from evolutionary and assay-labeled data. Nat Biotechnol 2022a;40:1114–22. 10.1038/S41587-021-01146-5 35039677

[vbag299-B30] Hsu C , Verkuil R, Liu J et al Learning inverse folding from millions of predicted structures. In: *Proceedings of the 39th International Conference on Machine Learning*, PMLR, Vol. 162, pp. 8946–70, 2022. 10.1101/2022.04.10.487779

[vbag299-B31] Jiang K , Yan Z, Bernardo M et al Rapid *in silico* directed evolution by a protein language model with EVOLVEpro. Science 2025;387:eadr6006. 10.1126/SCIENCE.ADR6006 39571002

[vbag299-B32] Jones EM , Lubock NB, Venkatakrishnan AJ et al Structural and functional characterization of G protein-coupled receptors with deep mutational scanning. Elife 2020;9:1–52. 10.7554/ELIFE.54895 PMC7707821 33084570

[vbag299-B33] Kolchina A , Dubanevics I, Kondrashov FA et al Beyond additivity: zero-shot methods cannot predict impact of epistasis on protein properties and function. bioRxiv, 10.64898/2026.02.17.706292, 6 July 2026, preprint: not peer reviewed.

[vbag299-B34] Laine E , Karami Y, Carbone A. GEMME: a simple and fast global epistatic model predicting mutational effects. Mol Biol Evol 2019;36:2604–19. 10.1093/MOLBEV/MSZ179 PMC6805226 31406981

[vbag299-B35] Lax BM , Escorial García M, Fernandez-Antunez C et al Enzymatic glucosylation enhances the solubility of niclosamide but abrogates its therapeutic efficacy. ACS Omega 2026;11:8667–77. 10.1021/ACSOMEGA.5C12185 PMC12902868 41696203

[vbag299-B36] Li M , Tan Y, Ma X et al ProSST: protein language modeling with quantized structure and disentangled attention. Adv Neural Inf Process Syst 2024a;37:35700–26. 10.52202/079017-1126

[vbag299-B37] Li M , Yu H, Fan G et al FS-Mutant: a few-shot learning benchmark for protein mutants mining. 2024b. Published online 28 February 2024. 10.21203/RS.3.RS-3987561/V1

[vbag299-B38] Lin Z , Akin H, Rao R et al Evolutionary-scale prediction of atomic-level protein structure with a language model. Science 2023;379:1123–30. 10.1126/SCIENCE.ADE2574 36927031

[vbag299-B39] Lutz S , Iamurri SM. Protein engineering: past, present, and future. Methods Mol Biol 2018;1685:1–12. 10.1007/978-1-4939-7366-8_1 29086300

[vbag299-B40] Mazurenko S , Prokop Z, Damborsky J. Machine learning in enzyme engineering. ACS Catal 2020;10:1210–23. 10.1021/ACSCATAL.9B04321

[vbag299-B41] Meier J , Rao R, Verkuil R et al Language models enable zero-shot prediction of the effects of mutations on protein function. Adv Neural Inf Process Syst 2021;34:29287–303.

[vbag299-B42] Mejia-Otalvaro F , Delima D, Mendonça C et al Stability engineering of sucrose synthase for robust UDP-glucose regeneration. bioRxiv, 10.64898/2026.01.30.702896, 31 January 2026, preprint: not peer reviewed.

[vbag299-B43] Mejia-Otalvaro F , Lax BM, Kırtel O et al Sustainable natural product glycosylation: a critical evaluation of biocatalytic and chemical approaches. ChemSusChem 2025;18:e202501094. 10.1002/CSSC.202501094 PMC12548952 40952024

[vbag299-B44] Nijkamp E , Ruffolo JA, Weinstein EN et al ProGen2: exploring the boundaries of protein language models. Cell Syst 2023;14:968–78.e3. 10.1016/j.cels.2023.10.002 37909046

[vbag299-B45] Notin P , Dias M, Frazer J et al Tranception: protein fitness prediction with autoregressive transformers and inference-time retrieval. In: *Proceedings of the 39th International Conference on Machine Learning*, PMLR, Vol. 28, June 2022a, pp. 16990–7017. https://proceedings.mlr.press/v162/notin22a.html (23 June 2025, date last accessed).

[vbag299-B46] Notin P , Kollasch A, Ritter D et al ProteinGym: large-scale benchmarks for protein fitness prediction and design. Adv Neural Inf Process Syst 2023;36:64331–79. 10.1101/2023.12.07.570727

[vbag299-B47] Notin P , Van Niekerk L, Kollasch AW et al TranceptEVE: combining family-specific and family-agnostic models of protein sequences for improved fitness prediction. bioRxiv, 10.1101/2022.12.07.519495, 27 December 2022b, preprint: not peer reviewed.

[vbag299-B48] Nutschel C , Fulton A, Zimmermann O et al Systematically scrutinizing the impact of substitution sites on thermostability and detergent tolerance for *Bacillus subtilis* lipase A. J Chem Inf Model 2020;60:1568–84. 10.1021/ACS.JCIM.9B00954 31905288

[vbag299-B49] Packer MS , Liu DR. Methods for the directed evolution of proteins. Nat Rev Genet 2015;16:379–94. 10.1038/NRG3927 26055155

[vbag299-B51] Raechell , Tee W-V, Dong B et al On the relationship between protein stability, thermostability, and allosteric signaling. J Mol Biol 2026;438:169537. 10.1016/j.jmb.2025.169537 41223935

[vbag299-B52] Rao R , Bhattacharya N, Thomas N et al Evaluating protein transfer learning with TAPE. Adv Neural Inf Process Syst 2019;32:9689–701. PMC7774645 33390682

[vbag299-B53] Rao RM , Liu J, Verkuil R et al MSA Transformer. Preprint, PMLR, 1 July 2021, 8844–56. https://proceedings.mlr.press/v139/rao21a.html (20 October 2025, date last accessed).

[vbag299-B54] Rives A , Meier J, Sercu T et al Biological structure and function emerge from scaling unsupervised learning to 250 million protein sequences. Proc Natl Acad Sci U S A 2021;118:e2016239118. 10.1073/PNAS.2016239118 PMC8053943 33876751

[vbag299-B55] Romero PA , Tran TM, Abate AR. Dissecting enzyme function with microfluidic-based deep mutational scanning. Proc Natl Acad Sci U S A 2015;112:7159–64. 10.1073/PNAS.1422285112 PMC4466731 26040002

[vbag299-B50] Notin P. Have We Hit the Scaling Wall for Protein Language Models? Substack. 7 May 2025. https://pascalnotin.substack.com/p/have-we-hit-the-scaling-wall-for (20 October 2025, date last accessed).

[vbag299-B56] Seuma M , Lehner B, Bolognesi B. An atlas of amyloid aggregation: the impact of substitutions, insertions, deletions and truncations on amyloid beta fibril nucleation. Nat Commun 2022;13:7084. 10.1038/S41467-022-34742-3 PMC9674652 36400770

[vbag299-B57] Sirirungruang S , Barnum CR, Tang SN et al Plant glycosyltransferases for expanding bioactive glycoside diversity. Nat Prod Rep 2023;40:1170–80. 10.1039/D2NP00077F 36853278

[vbag299-B58] Starr TN , Greaney AJ, Hilton SK et al Deep mutational scanning of SARS-CoV-2 receptor binding domain reveals constraints on folding and ACE2 binding. Cell 2020;182:1295–310.e20. 10.1016/j.cell.2020.08.012 PMC7418704 32841599

[vbag299-B59] Su J , Han C, Zhou Y et al SaProt: protein language modeling with structure-aware vocabulary. In: *International Conference on Representation Learning*, Vienna, Austria, 2024, pp. 6987–7009. (20 October 2025, date last accessed). 10.1101/2023.10.01.560349

[vbag299-B60] Tan Y , Wang R, Wu B et al From high-throughput evaluation to wet-lab studies: advancing mutation effect prediction with a retrieval-enhanced model. Bioinformatics 2025a;41:i401–9. 10.1093/BIOINFORMATICS/BTAF189 PMC12261477 40662802

[vbag299-B61] Tan Y , Zhou B, Zheng L et al Semantical and geometrical protein encoding toward enhanced bioactivity and thermostability. Elife 2025b;13:1–21. 10.7554/ELIFE.98033.4 PMC12048155 40314227

[vbag299-B62] Tee WV , Tan ZW, Lee K et al Exploring the allosteric territory of protein function. J Phys Chem B 2021;125:3763–80. 10.1021/ACS.JPCB.1C00540 33844527

[vbag299-B63] Teufel F , Kollasch AW, Huang Y et al Few-shot protein fitness prediction via in-context learning and test-time training. arXiv, http://arxiv.org/abs/2512.02315, 2 December 2025, preprint: not peer reviewed (31 March 2026, date last accessed).

[vbag299-B64] Tsishyn M , Hermans P, Rooman M et al Residue conservation and solvent accessibility are (almost) all you need for predicting mutational effects in proteins. Bioinformatics 2025;41:1–4. 10.1093/bioinformatics/btaf322 PMC12202862 40434923

[vbag299-B65] Visani GM , Verma A, DeWitt WS. Additive baselines furnish no evidence for epistasis learning by MULTI-evolve. bioRxiv, 10.64898/2026.04.23.719915, 24 April 2026, preprint: not peer reviewed.

[vbag299-B66] Weng C , Faure AJ, Escobedo A et al The energetic and allosteric landscape for KRAS inhibition. Nature 2024;626:643–52. 10.1038/s41586-023-06954-0 PMC10866706 38109937

[vbag299-B67] Wijma HJ , Floor RJ, Jekel PA et al Computationally designed libraries for rapid enzyme stabilization. Protein Eng Des Sel 2014;27:49–58. 10.1093/PROTEIN/GZT061 PMC3893934 24402331

[vbag299-B68] Wittmann BJ , Yue Y, Arnold FH. Informed training set design enables efficient machine learning-assisted directed protein evolution. Cell Syst 2021;12:1026–45.e7. 10.1016/j.cels.2021.07.008 34416172

[vbag299-B69] Woolley PR , Feller AL, Ellington AD et al Overestimating zero-shot fitness prediction: broad benchmarks mask local failures and practical limitations. bioRxiv, 10.64898/2026.06.04.730121, 25 June 2026, preprint: not peer reviewed.

[vbag299-B70] Yang KK , Fusi N, Lu AX. Convolutions are competitive with transformers for protein sequence pretraining. Cell Syst 2024;15:286–94.e2. 10.1016/J.CELS.2024.01.008 38428432

[vbag299-B71] Yang KK , Wu Z, Arnold FH. Machine-learning-guided directed evolution for protein engineering. Nat Methods 2019;16:687–94. 10.1038/S41592-019-0496-6 31308553

[vbag299-B72] Yang KK , Zanichelli N, Yeh H. Masked inverse folding with sequence transfer for protein representation learning. Protein Eng Des Sel 2023;36:1–10. 10.1093/PROTEIN/GZAD015 37883472

[vbag299-B73] Zhou Z , Zhang L, Yu Y et al Enhancing efficiency of protein language models with minimal wet-lab data through few-shot learning. Nat Commun 2024;15:5566. 10.1038/s41467-024-49798-6 PMC11219809 38956442

