# Supplementary material for: PRIZM: combining low-N data and zero-shot models to design enhanced protein variants
Source: Bioinform Adv. 2026 Oct 6;6(1):vbag299. doi: 10.1093/bioadv/vbag299 (PMC13760221; doi:10.1093/bioadv/vbag299)
Supplement: Supplementary file 1 [file vbag299_supplementary_data.zip › PRIZM_SI.pdf]

Supplementary Information

PRIZM: Combining Low-N Data and Zero-shot Models to Design Enhanced Protein Variants

David Harding-Larsen[1], Brianna M. Lax[1], Martina Escorial García[1], Catarina Mendonça[1], Felipe Mejia-Otalvaro[1], Ditte Hededam Welner[1,*], Stanislav Mazurenko[2,3,*]

[1] The Novo Nordisk Foundation Biotechnology Research Institute for the Green Transition, Søltofts Plads 220, 2200, Kongens Lyngby, Denmark

[2] Loschmidt Laboratories, Department of Experimental Biology and RECETOX, Faculty of Science, Masaryk University, Kamenice 5, 625 00, Brno, Czech Republic

[3] International Clinical Research Center, St. Anne's University Hospital, Pekařská 664/53, 602 00, Brno, Czech Republic

* Corresponding authors, diwel@dtu.dk (+45 93 51 34 98) & mazurenko@mail.muni.cz (+420 54949 4208)

Supplementary Methods

Case study 1

All materials and methods for Case Study 1, including protein expression, purification, and enzyme assays, were performed as described in Mejia-Otalvaro et al. (Mejia-Otalvaro *et al.* 2026), with identical buffer compositions and instruments. A brief summary is provided below.

Cloning and transformation

*Gm*SuSy-WT was obtained in a pET28a(+) expression vector containing an N-terminal His-tag and TEV protease cleavage site. Amino acid substitutions were introduced by PCR-based cloning, and all constructs were sequence-verified by Sanger sequencing prior to expression. Confirmed plasmids were transformed into chemically competent *E. coli* BL21 Star (DE3) cells.

Protein expression and purification

Expression strains were cultivated in kanamycin-supplemented rich medium at 37°C until an optical density at 600 nm (OD_{600}) between 0.5 and 0.8, followed by induction with isopropyl-β-D-thiogalactopyranoside (IPTG) and overnight protein expression at reduced temperature. Cells were harvested by centrifugation, resuspended in lysis buffer, and disrupted by sonication under cooling conditions. Clarified lysates were purified by Ni^{2+}-affinity chromatography using a HisTrap FF column on an ÄKTA pure system (Cytiva Life Sciences). Target protein fractions were identified by SDS-PAGE, pooled, buffer-exchanged, concentrated using 50 kDa centrifugal filters, aliquoted, and stored at −70°C. Protein concentrations were determined by UV absorbance at 280 nm.

Thermostability assays

Protein thermodynamic stability was evaluated by determining apparent melting temperatures ($T_{m,app}$) using differential scanning fluorimetry (DSF) through Protein Thermal Shift Dye kit. Fluorescence-based unfolding profiles were recorded during controlled heating using a QuantStudio 5 real-time PCR system (Applied Biosystems). Measurements were performed with four technical replicates.

Residual activity assays

Residual enzymatic activity as a proxy for kinetic stability was assessed following short-term thermal treatment of protein samples prior to reaction initiation. Enzymatic activity was quantified using a sucrose/UDP-based assay, and fructose formation was measured with a commercial enzymatic detection kit (Megazyme). Residual activity was calculated relative to the activity of the thermal untreated wild-type enzyme and used as a measure of kinetic stability. All measurements were performed in triplicate.

Case study 2

All materials and methods for Case Study 2, including protein expression, purification, and enzyme assays, were performed as described in Lax et al. (Lax *et al.* 2026), with identical buffer compositions and instruments. A brief summary is provided below.

Cloning and transformation

The WT TOGT1_1 gene was previously ordered with in a pET28a(+) expression vector containing N- and C-terminal His-tags and a TEV protease cleavage site from Biomatik(Harding-Larsen *et al.* 2024). All amino acid substitutions introduced by PCR-based site-directed mutagenesis, and all constructs were sequence-verified by Sanger sequencing prior to expression. Confirmed plasmids were transformed into chemically competent *E. coli* BL21 Star (DE3) cells.

Protein expression and purification

Expression strains were cultivated in kanamycin-supplemented rich medium at 37°C until reaching an OD_{600} of 0.8-1, followed by induction with 250 µM IPTG and overnight protein expression at reduced temperature. Cells were harvested by centrifugation, resuspended in lysis buffer, and disrupted by sonication under cooling conditions. Clarified lysates were purified by Ni^{2+}-affinity chromatography using a HisTrap FF column on an ÄKTA pure system (Cytiva Life Sciences). Target protein fractions were identified by SDS-PAGE, pooled, buffer-exchanged, concentrated using 50 kDa centrifugal filters, aliquoted, and stored at −70°C. Protein concentrations were determined by UV absorbance at 280 nm.

Enzyme activity assays

The TOGT1_1 glycosylation reactions were measured using purified protein (50 µg/mL), Nic/Nic-Glc (200 µM), and UDP-Glc (5 mM) in 100 µL reaction buffer and 20% (v/v) DMSO. All reactions were incubated at 30°C, quenched with equivolume methanol, filtered, and analyzed by reversed-phase HPLC (Ultimate 3000 Series HPLC, Thermo Fisher Scientific). HPLC analysis was performed using a ZORBAX RR Eclipse Plus C18 column (100 × 4.6 mm, 3.5 µm; Agilent) at 30°C. Mobile phases were water + 0.1% formic acid (A) and acetonitrile (B), with a 1 mL/min gradient elution following the method described in Lax et al.(Lax *et al.* 2026). Chromatograms were recorded at 330 nm using Chromeleon 7.2.9, and relative activities were determined from integrated peak areas.

Guidance for using PRIZM

Here, we provide practical guidance on when and how to implement PRIZM most effectively.

Regarding when to leverage the workflow for variant nomination, three settings generally favor the use of PRIZM. The first is the presence of a mutational dataset from prior engineering campaigns. Unlike several state-of-the-art methods (Zhou *et al*. 2024, Jiang *et al*. 2025, Teufel *et al*. 2025), PRIZM is designed to work with any existing mutational dataset, and we therefore encourage researchers to leverage PRIZM together with datasets from previous campaigns. The second setting favoring PRIZM is when budget restrictions allow for only a few rounds of experiments, as this will reduce the effectiveness of few-shot modelling approaches such as EVOLVEpro (Jiang *et al*. 2025). The PRIZM rankings do not depend on multiple rounds of experiments and can thus be utilized as a single-round nominator. Lastly, researchers with limited ML expertise might also benefit from using PRIZM instead of more elaborate supervised strategies that require model fine-tuning, hyperparameter optimization, and careful train-test splitting. Conversely, when no prior experimental data exists and budget allows for multiple iterative rounds, supervised or few-shot approaches are likely to achieve higher predictive accuracy and may be preferred. Here, we suggest using PRIZM to identify the best initial model for subsequent fine-tuning.

When running PRIZM, it is important to evaluate the performance metrics of the best models, as the random baseline of these metrics are influenced by both dataset size and bias (Figure S5-6). If the best model is worse than a random baseline, its predictions may not reliably capture mutational effects. As PRIZM can never be better than the best model, we do not advise the use of PRIZM in such a scenario.

When choosing a threshold value for defining low and high-performing variants, we advise using the WT value, as this will naturally divide the data into variants better and worse than the WT. However, as the PRIZM model rankings were found to be robust across a range of binarization thresholds, the specific threshold should not substantially alter the general rankings (Figure S7).

Our retrospective DMS benchmarking demonstrated that PRIZM can robustly distinguish between high- and low-performing models using as few as 20 variants. For any dataset sizes below this, we advise caution when utilizing the workflow. Although PRIZM successfully identified improved TOGT1_1 variants using only 8 initial mutants, we incorporated expert evaluation in addition to the model rankings. An expert curation of the PRIZM results can thus be a potential avenue for utilizing the workflow in very low-N settings. Generally, we chose to incorporate an additional curation step for low-N datasets below 50 variants, though this is not a strict cutoff.

For low-N datasets of 50 or more variants, the best models identified by PRIZM generally provide reliable guidance for selecting new variants. If one model clearly outperforms all other models, we recommend using only this model for ranking the *in silico* library. However, if several models exhibit similar performance, our results suggest utilizing multiple models for variant selection. This can either be implemented using a simple additive combination of the variant ranks, as demonstrated in our *Gm*SuSy case study, or by more elaborate ensemble strategies such as that proposed by Hie et al. (Hie *et al.* 2024). We suggest mainly combining models based on different protein information types, to reduce the potential for redundant information.

Finally, when selecting the variants for further experimental characterization, we recommend balancing exploitation (testing the highest-ranked candidates) with exploration (sampling diverse regions of sequence space). Although some substitutions may appear nonsensical from the perspective of domain expertise, discarding such variants outright contradicts the motivation for using zero-shot models, which is to identify beneficial mutations that lie outside conventional domain knowledge. Conversely, some model rankings may be dominated by mutations at a single position and prioritizing only these can result in a narrow search of the mutational landscape. To avoid this, we suggest selecting the top-ranked variants while ensuring coverage across multiple positions. If there are a large number of positions with high apparent mutability, the model rankings can also be used for the design of smart libraries.

Supplementary Figures

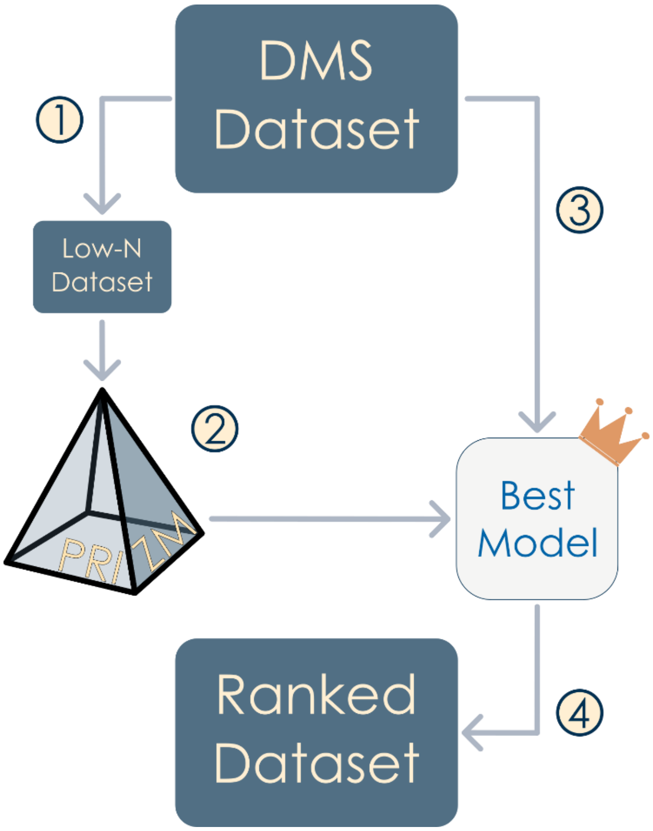

Figure S1 – Validation approach for PRIZM using low-N datasets from full DMS datasets. 1) Subsampling of full DMS datasets from ProteinGym (Notin *et al.* 2023) to generate low-N dataset; 2) identification of the best model for low-N dataset; 3) processing of full DMS dataset using best model; 4) ranking of DMS dataset using zero-shot scores and subsequent performance evaluation.

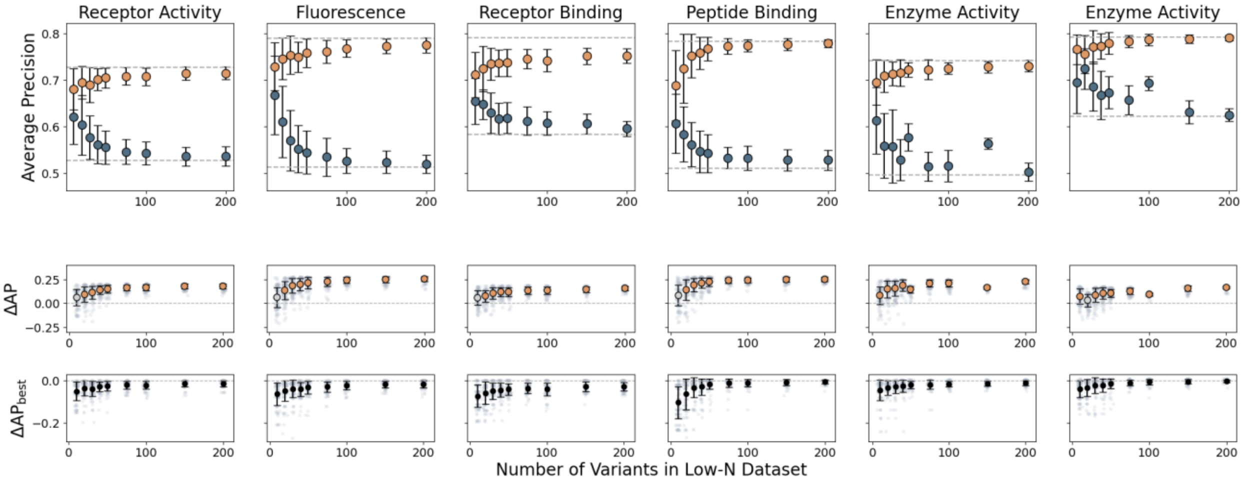

Figure S2 – Performance of zero-shot models (average precision) selected by PRIZM as a function of the number of variants in the low-N dataset. Figure shows performance on full DMS datasets, with receptor activity from Jones et al. (Jones *et al.* 2020), fluorescence data from Ellis et al. (Ellis *et al.* 2024), receptor binding from Starr et al. (Starr *et al.* 2020), peptide binding from Araya et al. (Araya *et al.* 2012), and enzyme activity from Romero et al. (Romero, Tran, and Abate 2015) and Chiasson et al. (Chiasson *et al.* 2020). The top row shows average precision (AP) for the best (orange) and worst (blue) models, with dotted lines indicating the zero-shot upper and lower limit on the whole dataset. The middle row shows ΔAP between the best and worst models, colored by Cohen's d (blue: $d < 0.2$, grey: $d < 0.5$, orange: $d > 0.5$). The bottom row shows ΔAP between each selected model and the zero-shot upper limit on the entire dataset (ΔAP_{best}). Error bars represent standard deviation from 100 PRIZM iterations.

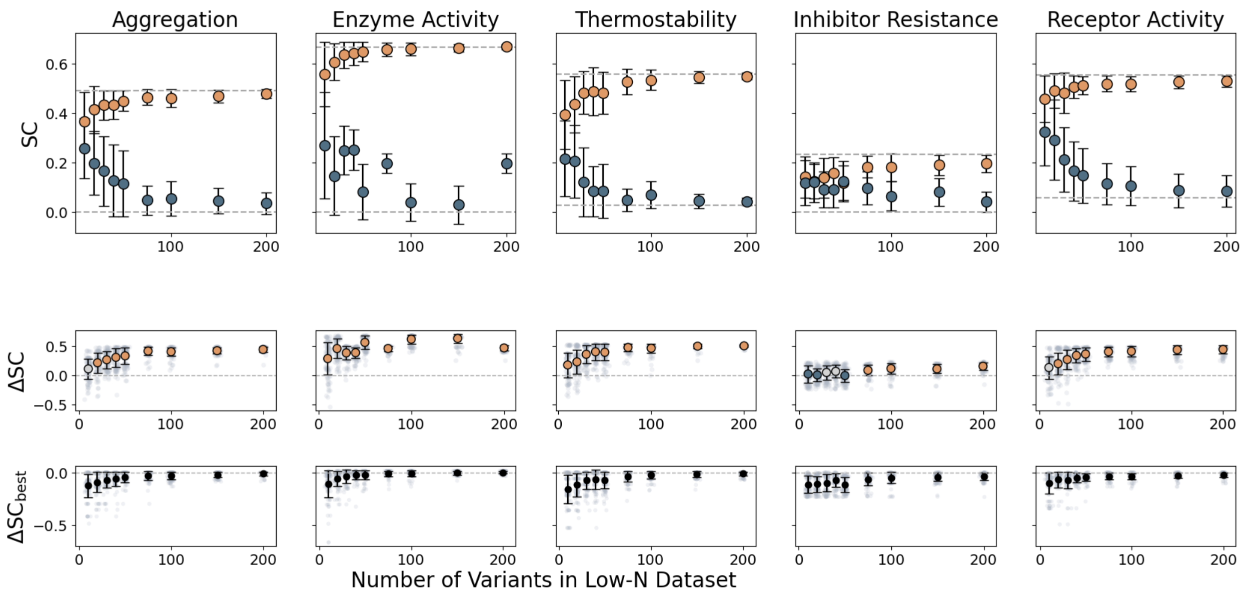

Figure S3 – Performance of zero-shot models (Spearman correlation) selected by PRIZM as a function of the number of variants in the low-N dataset. Performance of zero-shot models selected by PRIZM for predicting full DMS datasets as a function of the number of variants in the low-N dataset, with aggregation data from Seuma et al. (Seuma, Lehner, and Bolognesi 2022), enzyme activity from Hobbs et al. (Hobbs *et al.* 2022), thermostability from Nutschel et al. (Nutschel *et al.* 2020), inhibitor resistance from Brenan et al. (Brenan *et al.* 2016)), and receptor activity from Jones et al. (Jones *et al.* 2020). The top row shows Spearman correlation (SC) for the best (orange) and worst (blue) models, with dotted lines indicating the zero-shot upper and lower limit on the whole dataset. The middle row shows ΔSC between the best and worst models, colored by Cohen's d (blue: $d < 0.2$, grey: $d < 0.5$, orange: $d > 0.5$). The bottom row shows ΔSC between each selected model and the zero-shot upper limit on the entire dataset (ΔSC_{best}). Error bars represent standard deviation from 100 PRIZM iterations.

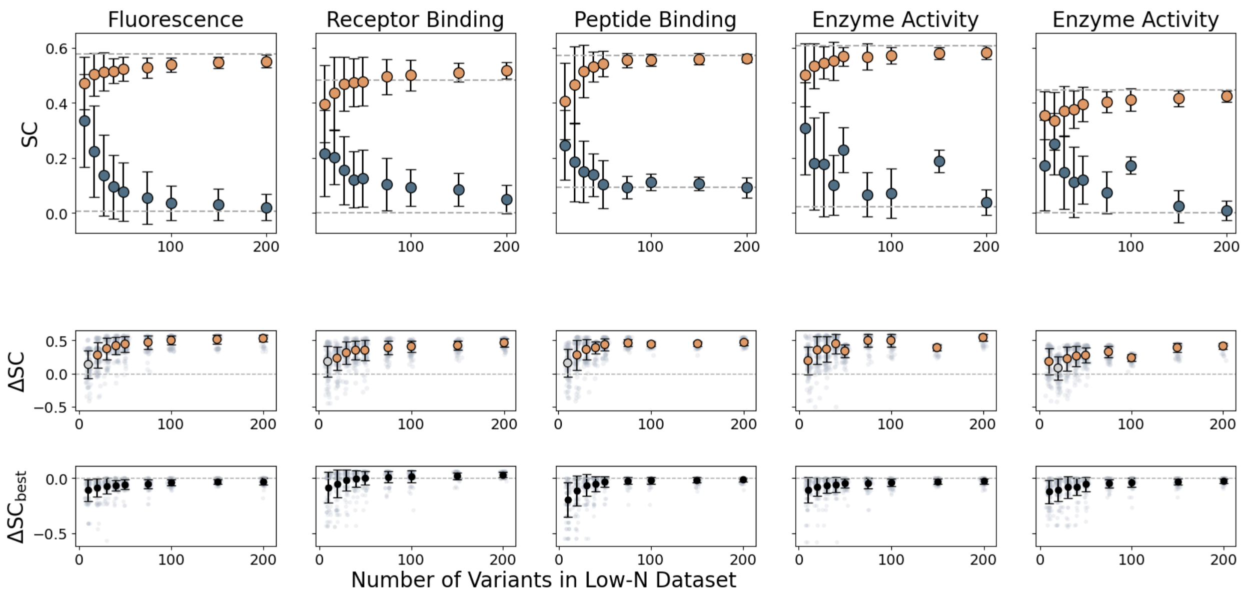

Figure S4 – Performance of zero-shot models (Spearman correlation) selected by PRIZM as a function of the number of variants in the low-N dataset. Performance of zero-shot models selected by PRIZM for predicting full DMS datasets as a function of the number of variants in the low-N dataset with fluorescence data from Ellis et al. (Ellis *et al*. 2024), receptor binding from Starr et al. (Starr *et al*. 2020), peptide binding from Araya et al. (Araya *et al*. 2012), and enzyme activity from Romero et al. (Romero, Tran, and Abate 2015) and Chiasson et al. (Chiasson *et al*. 2020). The top row shows Spearman correlation (SC) for the best (orange) and worst (blue) models, with dotted lines indicating the zero-shot upper and lower limit on the whole dataset. The middle row shows ΔSC between the best and worst models, colored by Cohen's d (blue: d < 0.2, grey: d < 0.5, orange: d > 0.5). The bottom row shows ΔSC between each selected model and the zero-shot upper limit on the entire dataset (ΔSC_{best}). Error bars represent standard deviation from 100 PRIZM iterations.

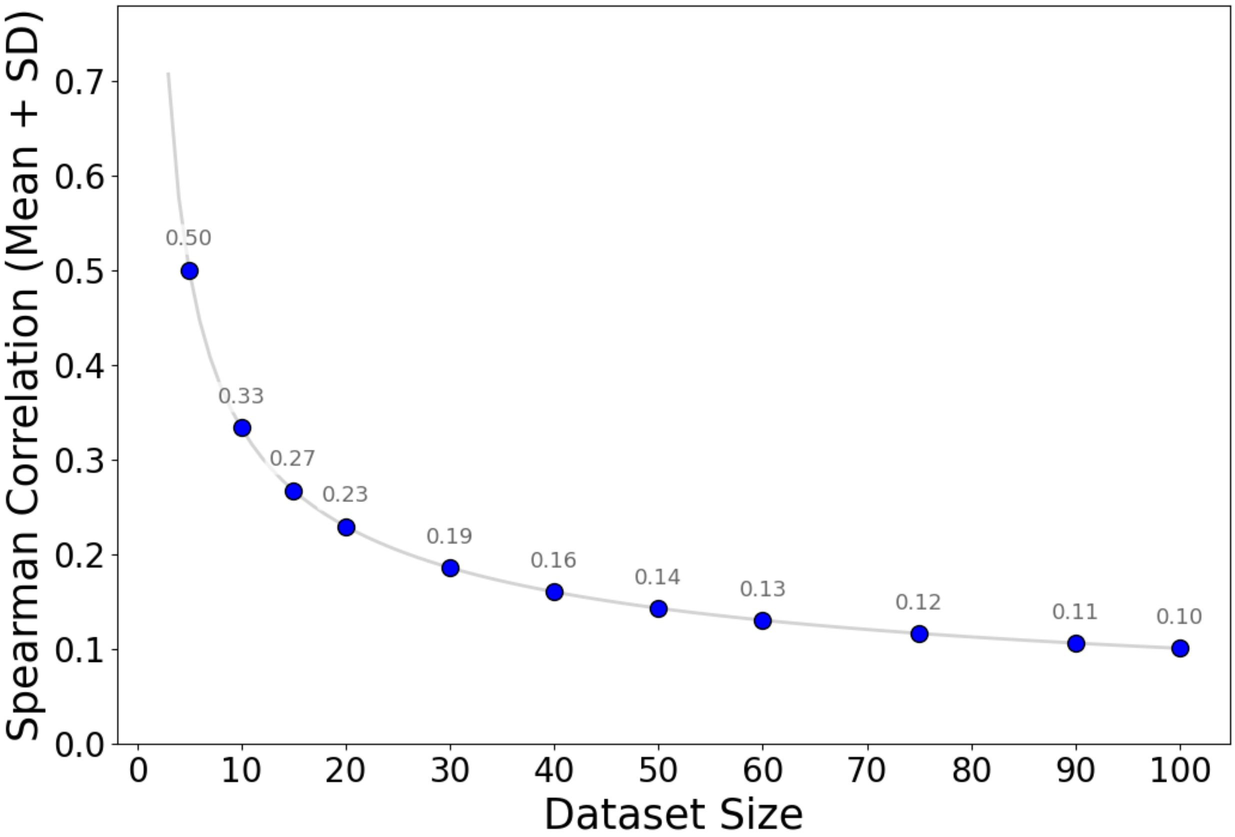

Figure S5 – Spearman correlation as a function of dataset size. Spearman correlation floor (mean plus one standard deviation) for a random predictor as a function of dataset size, computed from the closed form $1/\sqrt{N-1}$, see (Hotelling and Pabst 1936). The gray line denotes the continuous floor, while the blue points mark specific dataset sizes of 5, 10, 15, 20, 30 40, 50, 60, 75, 90, and 100..

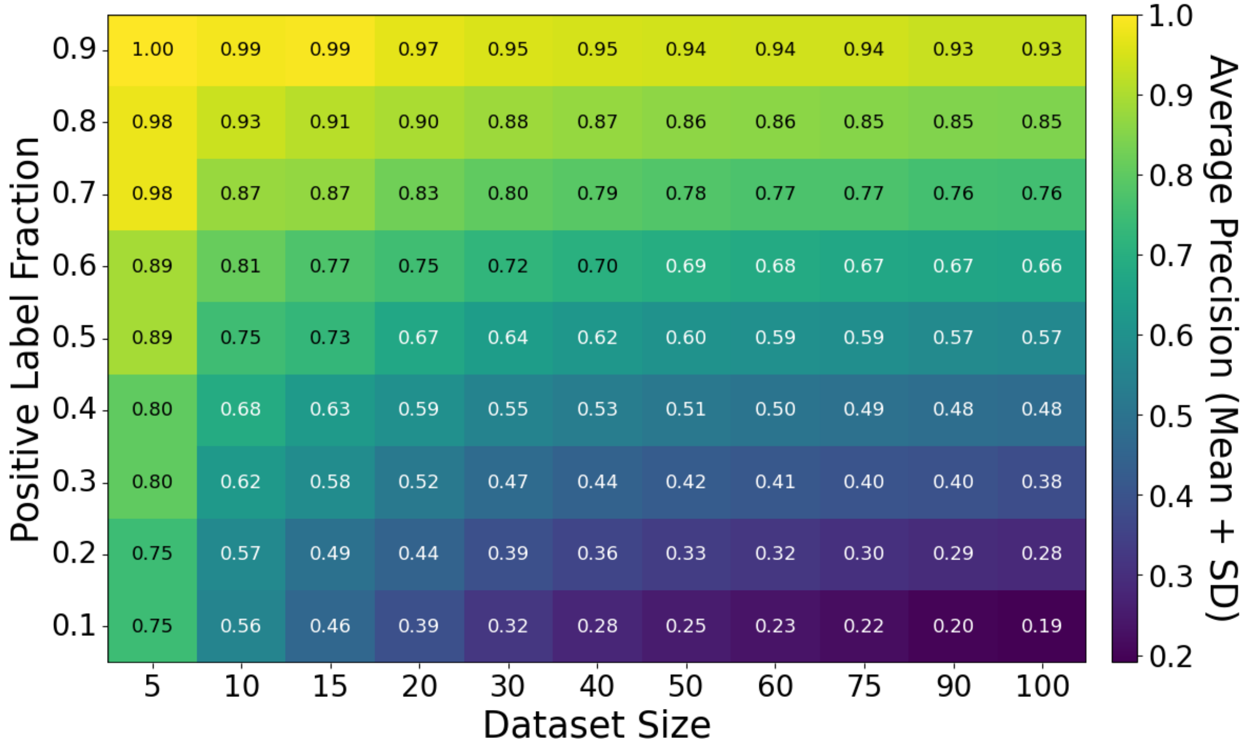

Figure S6 – Average precision as a function of dataset size and positive label fraction. Average precision floor (mean plus one standard deviation) of a random predictor for different dataset sizes and positive label fractions, denoted with both color and value. Values are computed exactly from closed-form expressions for the mean and variance of average precision under a uniformly random ranking from (Manzhos, Ianevych, and Melnyk 2026).

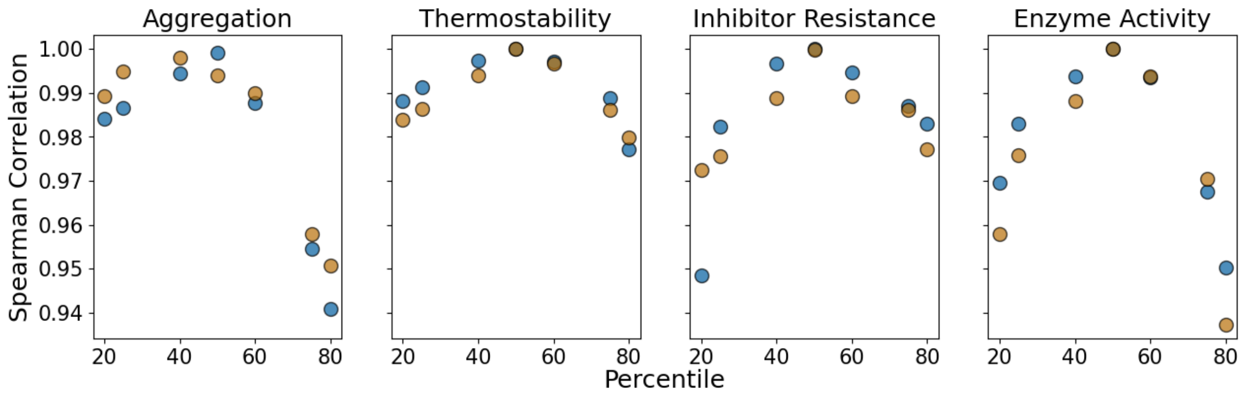

Figure S7 — Sensitivity of model ranking to binarization threshold. Spearman correlation between model rankings obtained using percentile-based thresholds and rankings obtained using the default threshold from ProteinGym (Notin *et al.* 2023), shown for four representative benchmark datasets (Brenan *et al.* 2016, Nutschel *et al.* 2020, Hobbs *et al.* 2022, Seuma, Lehner, and Bolognesi 2022). Blue points represent model rankings for the full dataset, while orange points denote average rankings for 100 iterations of subsampling of 50 variants.

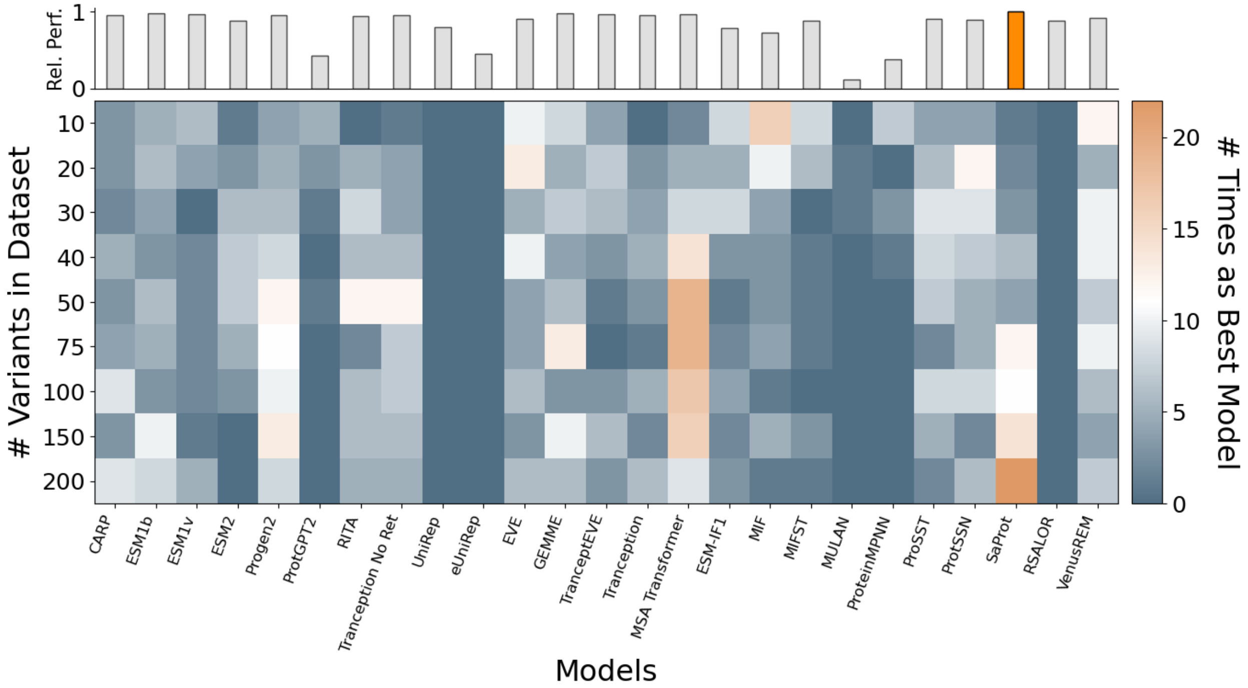

Figure S8 – Frequency of each model being selected as the best by PRIZM for receptor activity from Jones et al. (Jones *et al.* 2020)**.** The figure shows data from 100 iterations of the PRIZM workflow. The top inset shows each model's average precision relative to the overall best model (orange) on the full dataset.

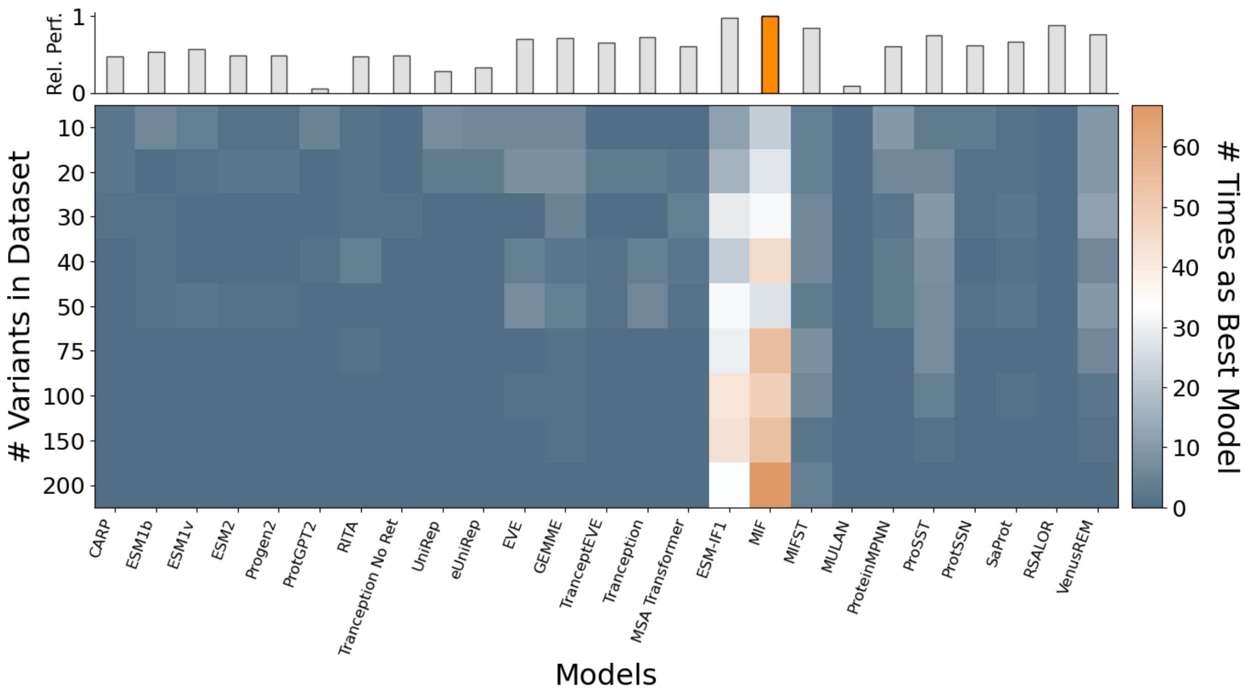

Figure S9 – Frequency of each model being selected as the best by PRIZM for thermostability from Nutschel et al. (Nutschel *et al*. 2020)**.** The figure shows data from 100 iterations of the PRIZM workflow. The top inset shows each model's average precision relative to the overall best model (orange) on the full dataset.

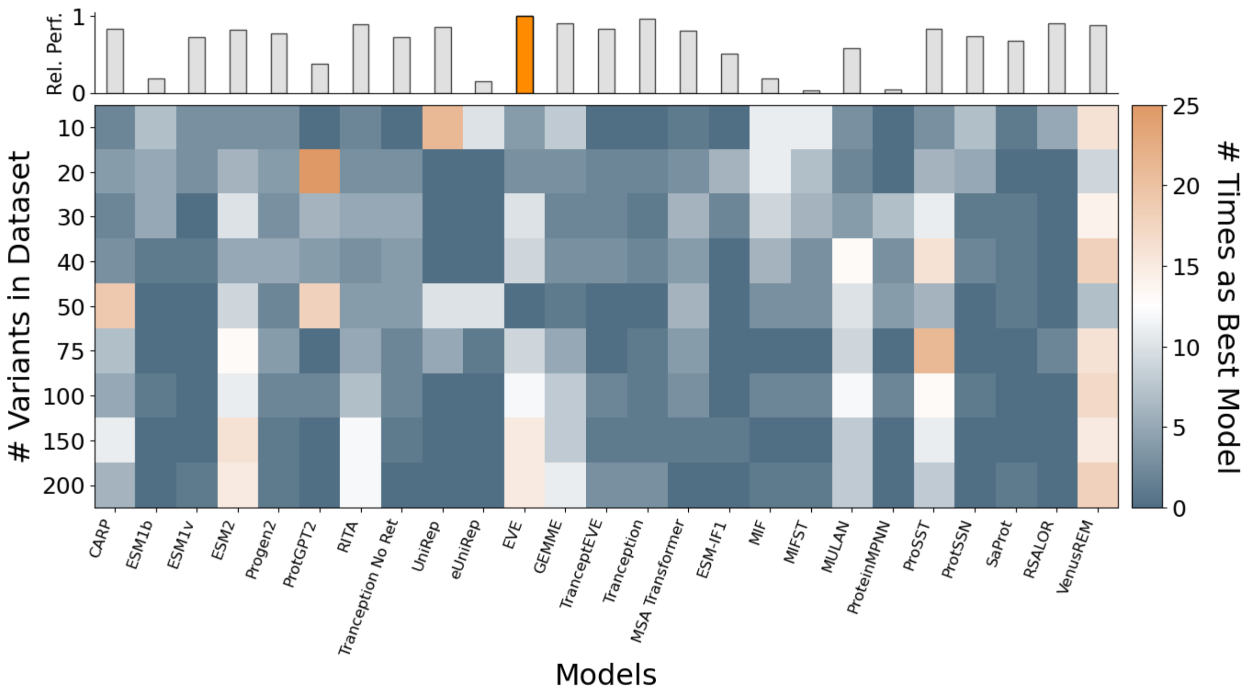

Figure S10 – Frequency of each model being selected as the best by PRIZM for inhibitor resistance from Brenan et al. (Brenan *et al.* 2016)**.** The figure shows data from 100 iterations of the PRIZM workflow. The top inset shows each model's average precision relative to the overall best model (orange) on the full dataset.

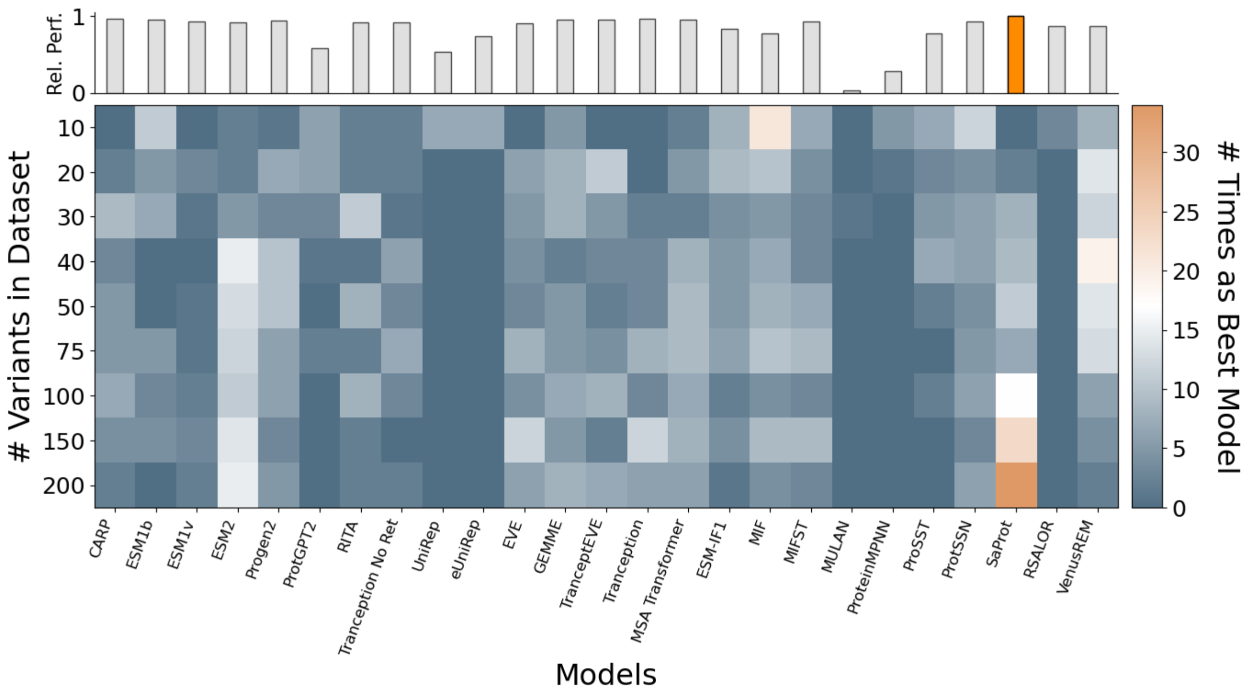

Figure S11 – Frequency of each model being selected as the best by PRIZM for fluorescence from Ellis et al. (Ellis *et al.* 2024)**.** The figure shows data from 100 iterations of the PRIZM workflow. The top inset shows each model's average precision relative to the overall best model (orange) on the full dataset.

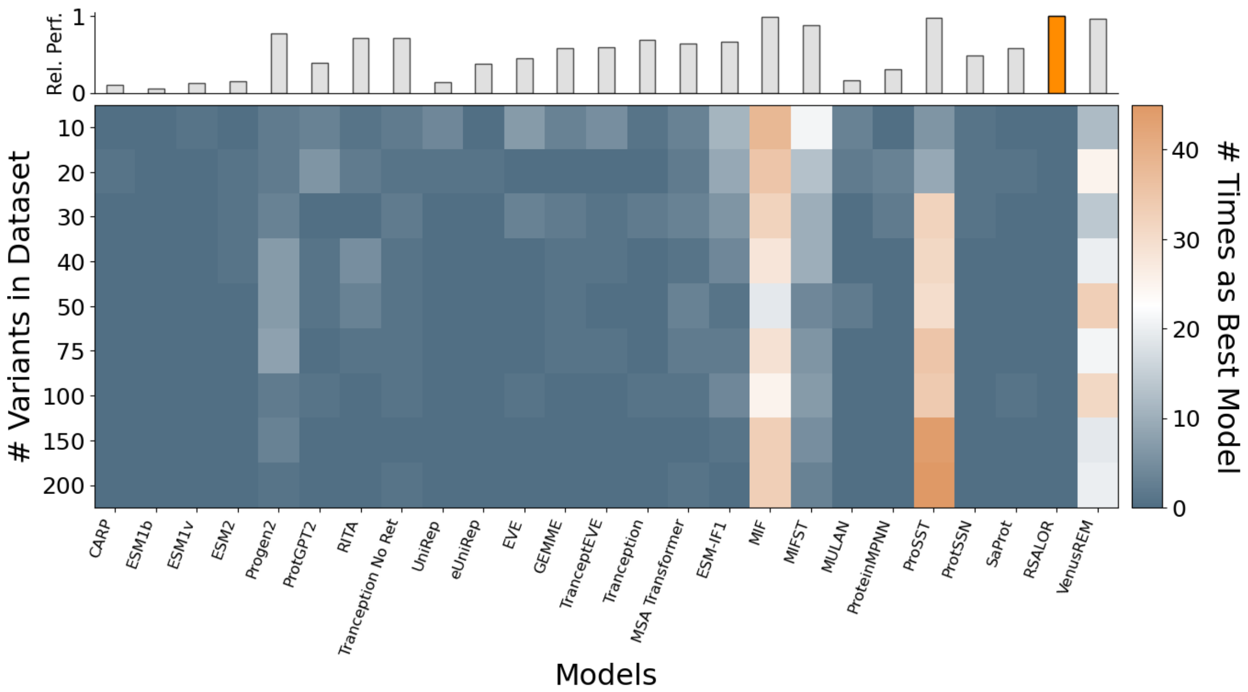

Figure S12 – Frequency of each model being selected as the best by PRIZM for receptor binding from Starr et al. (Starr *et al.* 2020)**.** The figure shows data from 100 iterations of the PRIZM workflow. The top inset shows each model's average precision relative to the overall best model (orange) on the full dataset.

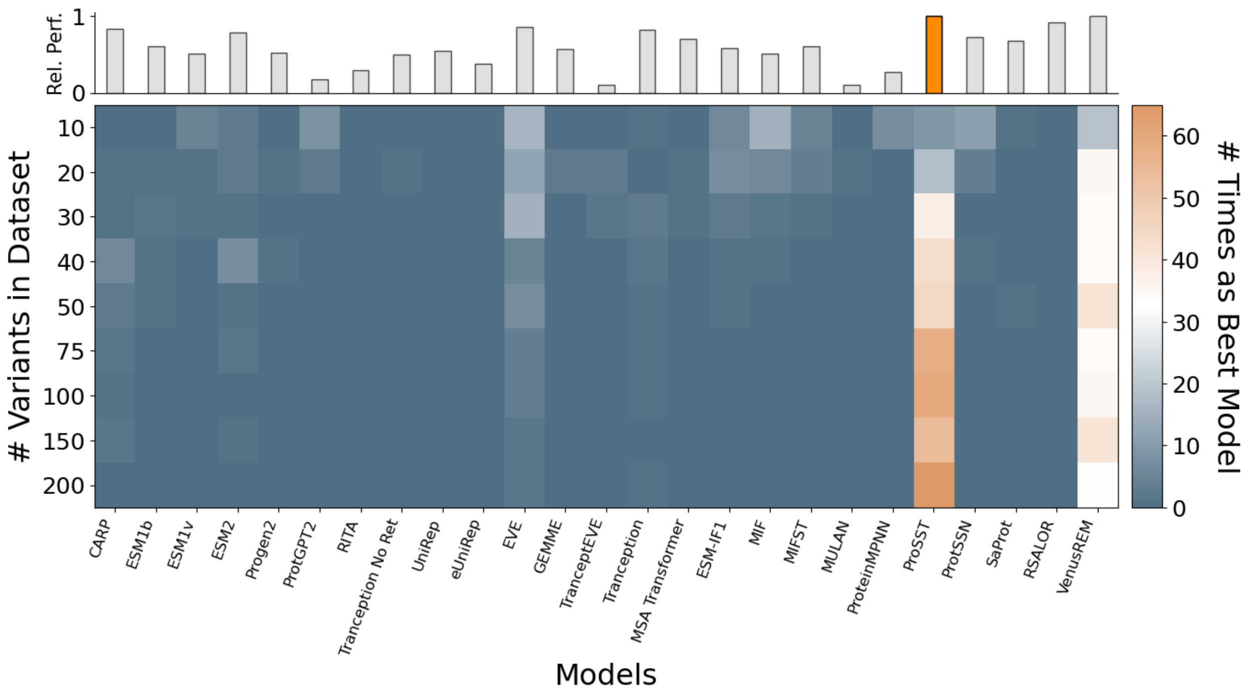

Figure S13 – Frequency of each model being selected as the best by PRIZM for peptide binding from Araya et al. (Araya *et al.* 2012)**.** The figure shows data from 100 iterations of the PRIZM workflow. The top inset shows each model's average precision relative to the overall best model (orange) on the full dataset.

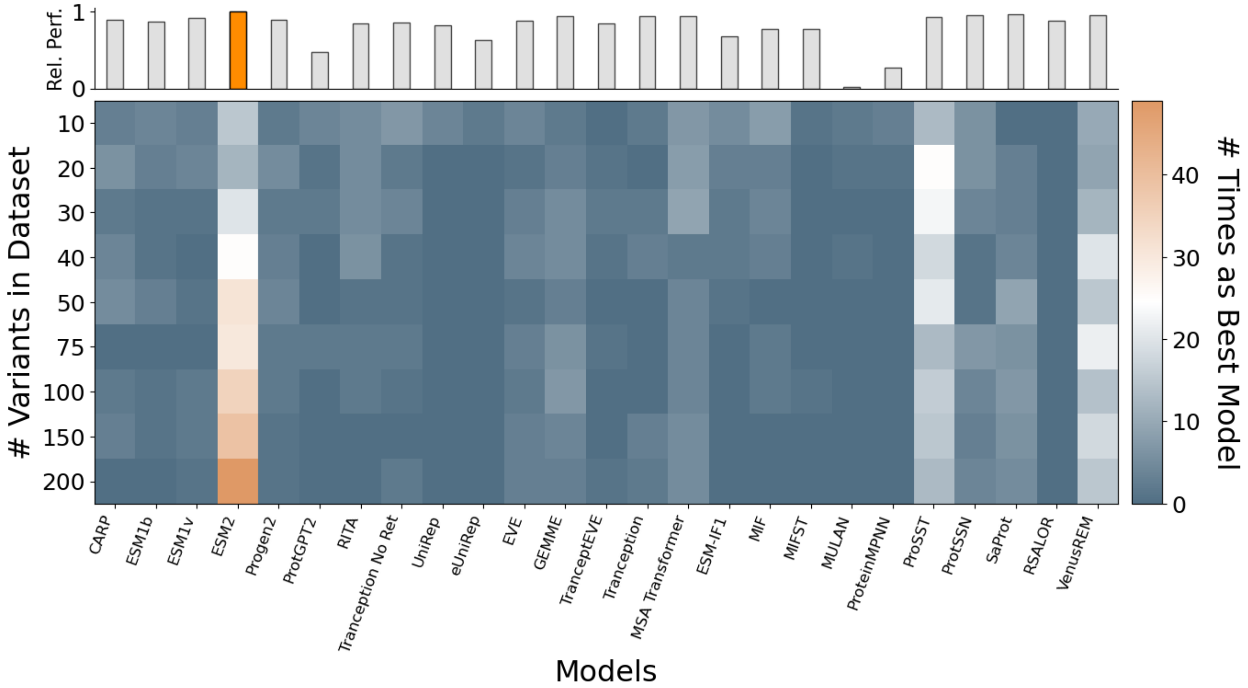

Figure S14 – Frequency of each model being selected as the best by PRIZM for peptide binding from Hobbs et al. (Hobbs *et al.* 2022)**.** The figure shows data from 100 iterations of the PRIZM workflow. The top inset shows each model's average precision relative to the overall best model (orange) on the full dataset.

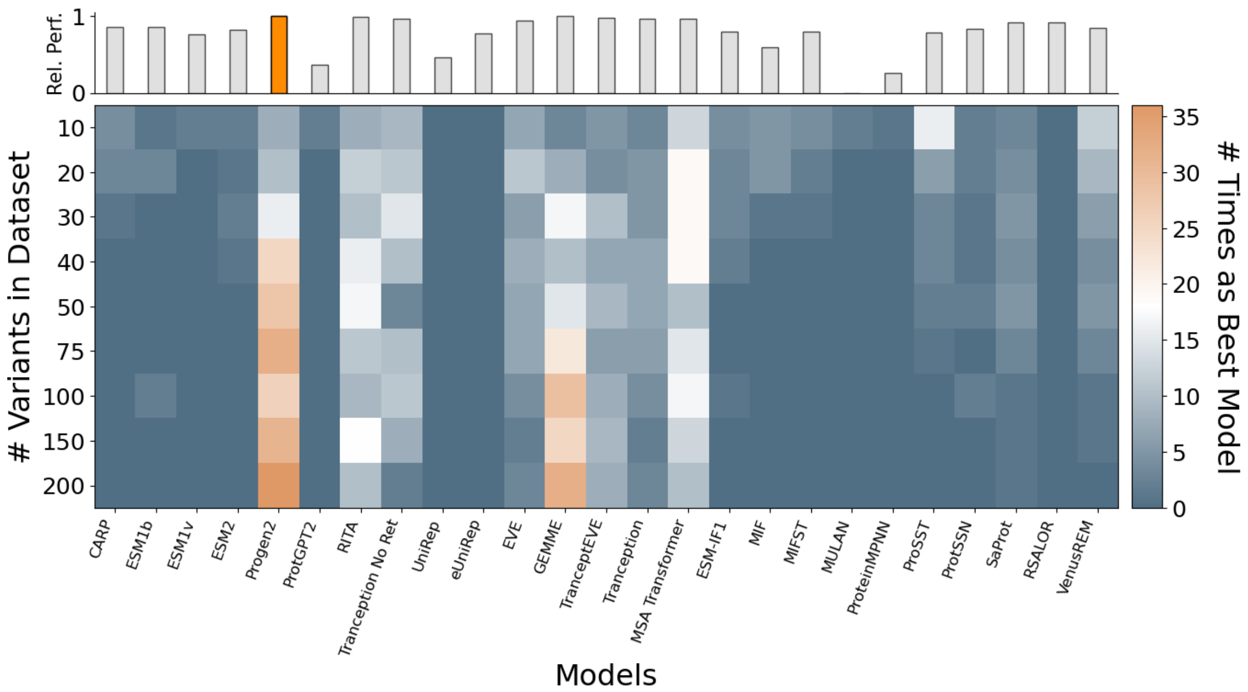

Figure S15 – Frequency of each model being selected as the best by PRIZM for peptide binding from Romero et al. (Romero, Tran, and Abate 2015)**.** The figure shows data from 100 iterations of the PRIZM workflow. The top inset shows each model's average precision relative to the overall best model (orange) on the full dataset.

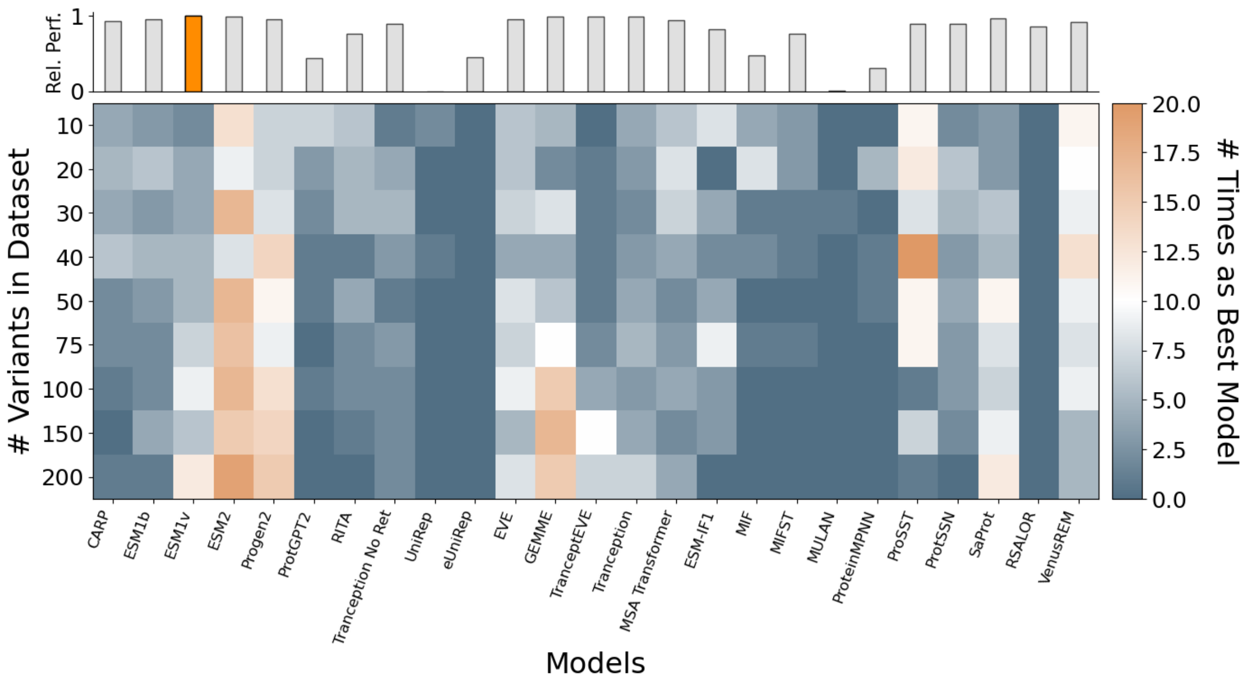

Figure S16 – Frequency of each model being selected as the best by PRIZM for peptide binding from Chiasson et al. (Chiasson *et al.* 2020)**.** The figure shows data from 100 iterations of the PRIZM workflow. The top inset shows each model's average precision relative to the overall best model (orange) on the full dataset.

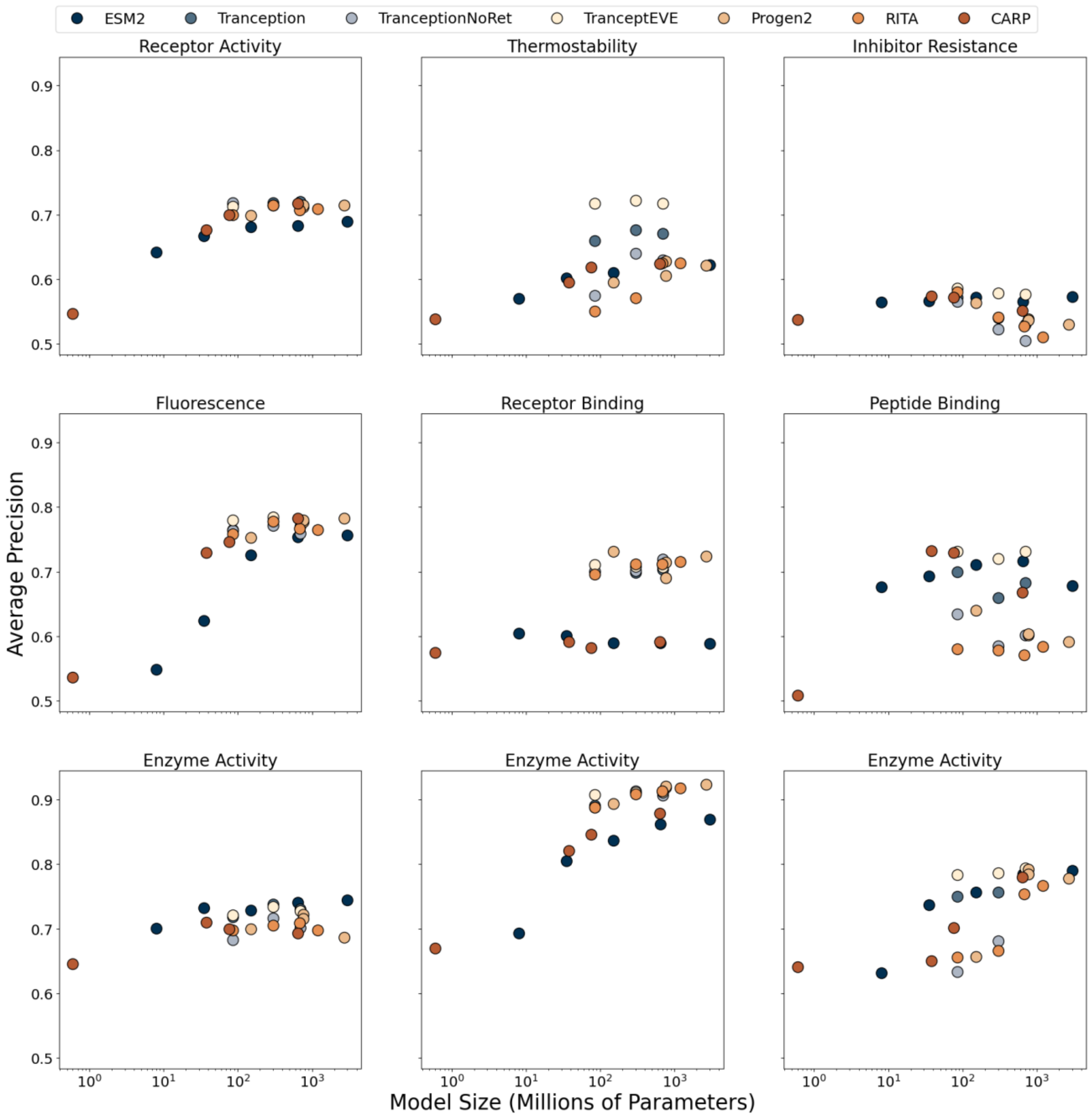

Figure S17 – Average precision of models as a function of model size. Figure shows performance on full DMS datasets, with receptor activity data from Jones et al. (Jones *et al.* 2020), thermostability from Nutschel et al. (Nutschel *et al.* 2020), inhibitor resistance from Brenan et al. (Brenan *et al.* 2016) , fluorescence data from Ellis et al. (Ellis *et al.* 2024), receptor binding from Starr et al. (Starr *et al.* 2020), peptide binding from Araya et al. (Araya *et al.* 2012), and enzyme activity from Hobbs et al. (Hobbs *et al.* 2022), Romero et al. (Romero, Tran, and Abate 2015), and Chiasson et al. (Chiasson *et al.* 2020).

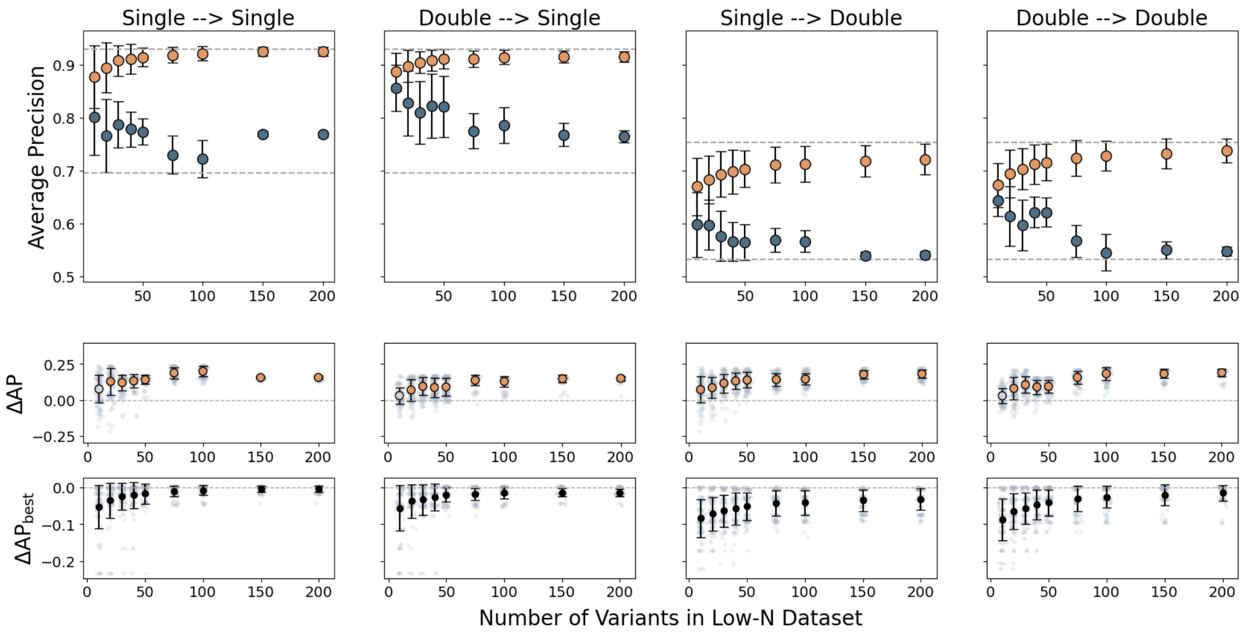

Figure S18 – Performance of zero-shot models selected by PRIZM using single or double mutants for predicting aggregation data (Seuma, Lehner, and Bolognesi 2022)**.** "Single → Double" indicates Model Selection using only single mutants and Variant Ranking evaluation on double mutants; other combinations follow the same scheme. The top row shows average precision (AP) for the best (orange) and worst (blue) models, with dotted lines indicating the overall best and worst models. The middle row shows ΔAP between the best and worst models, colored by Cohen's d (blue: d < 0.2, grey: d < 0.5, orange: d > 0.5). The bottom row shows ΔAP between each selected model and the best overall model (ΔAP_{best}). Error bars represent standard deviation from 100 PRIZM iterations.

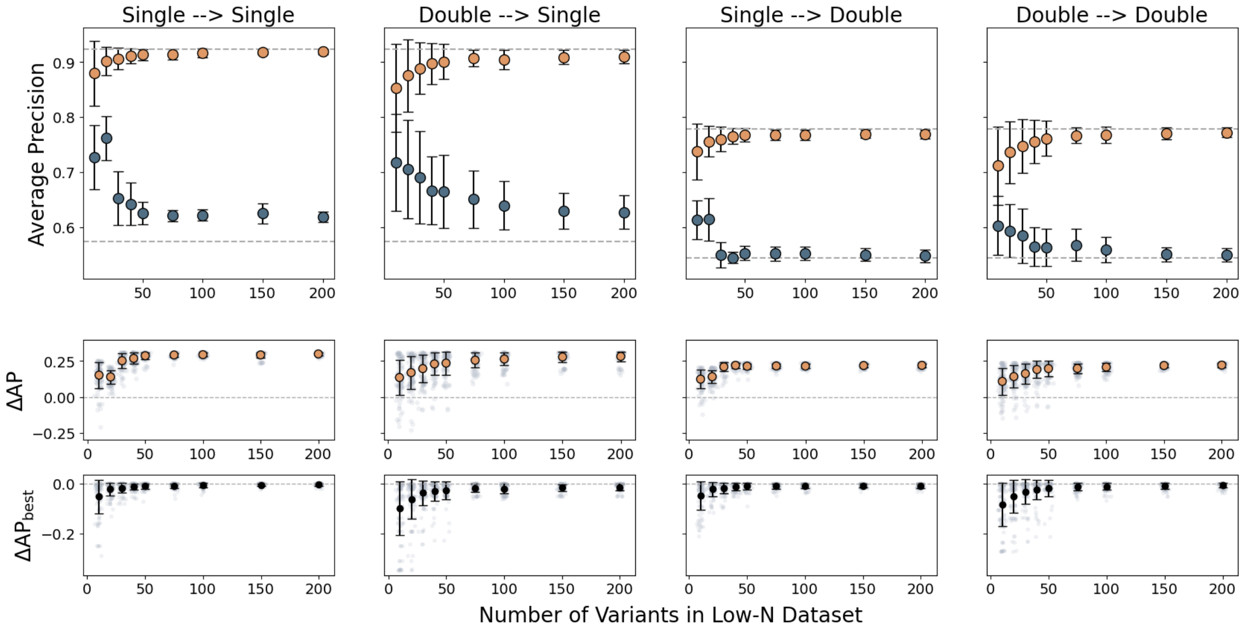

Figure S19 – Performance of zero-shot models selected by PRIZM using single or double mutants for predicting peptide binding data (Araya *et al.* 2012)**.** "Single → Double" indicates Model Selection using only single mutants and Variant Ranking evaluation on double mutants; other combinations follow the same scheme. The top row shows average precision (AP) for the best (orange) and worst (blue) models, with dotted lines indicating the overall best and worst models. The middle row shows ΔAP between the best and worst models, colored by Cohen's d (blue: $d < 0.2$, grey: $d < 0.5$, orange: $d > 0.5$). The bottom row shows ΔAP between each selected model and the best overall model (ΔAP_{best}). Error bars represent standard deviation from 100 PRIZM iterations.

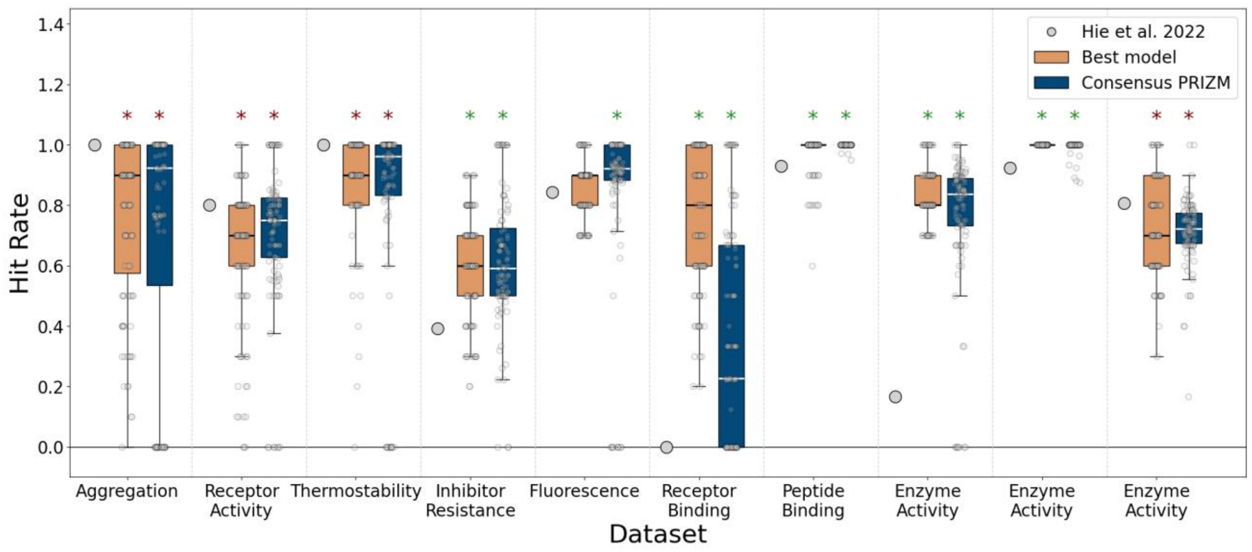

Figure S20 – Hit rate of best models identified by PRIZM compared to the approach by Hie et al. (Hie *et al.* 2024)**.** Box plots show the 25th, 50th (median), and 75th percentiles from 100 PRIZM iterations using 50 variants. Gray circles indicate individual hit rates for each method. Asterisks denote significant differences (p = 0.0001) based on a one-sided Mann–Whitney U test (green = PRIZM significantly better than Hie et al.; red = significantly worse).

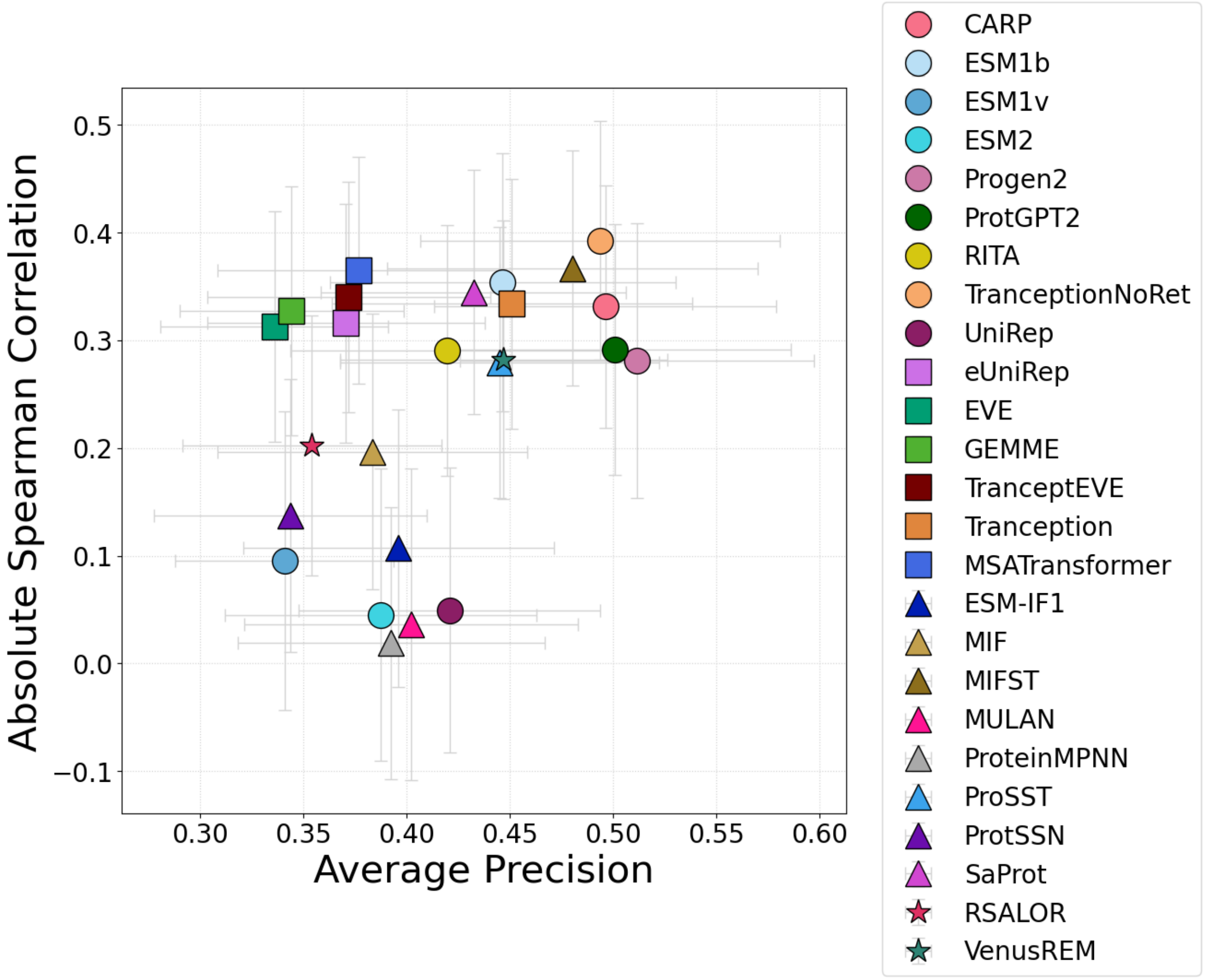

Figure S21 – Performance metrics for the PRIZM zero-shot models when predicting *Gm*SuSy $T_{m,\ app}$. Circle = sequence-based model, square = MSA-based model, triangle = structure-based model, star = all three. Error bars indicate the standard deviation using a bootstrapping approach with N = 1000.

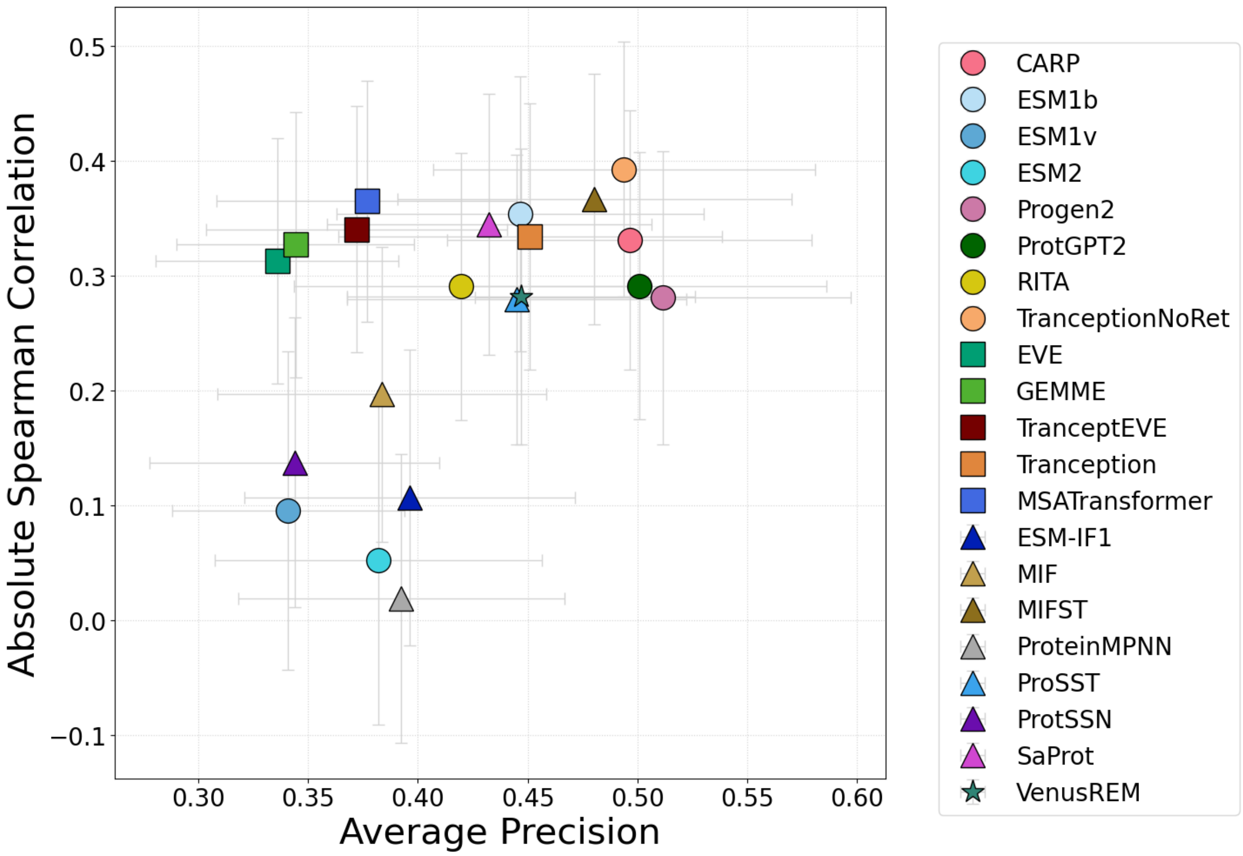

Figure S22 – Performance metrics for the PRIZM zero-shot models with correct direction when predicting *Gm*SuSy $T_{m,\ app}$. Only models predicting the correct direction of the mutational effect (e.g., higher values corresponding to improved variants) are shown. Circle = sequence-based model, square = MSA-based model, triangle = structure-based model, star = all three. Error bars indicate the estimated standard deviation using a bootstrapping approach (N = 1000).

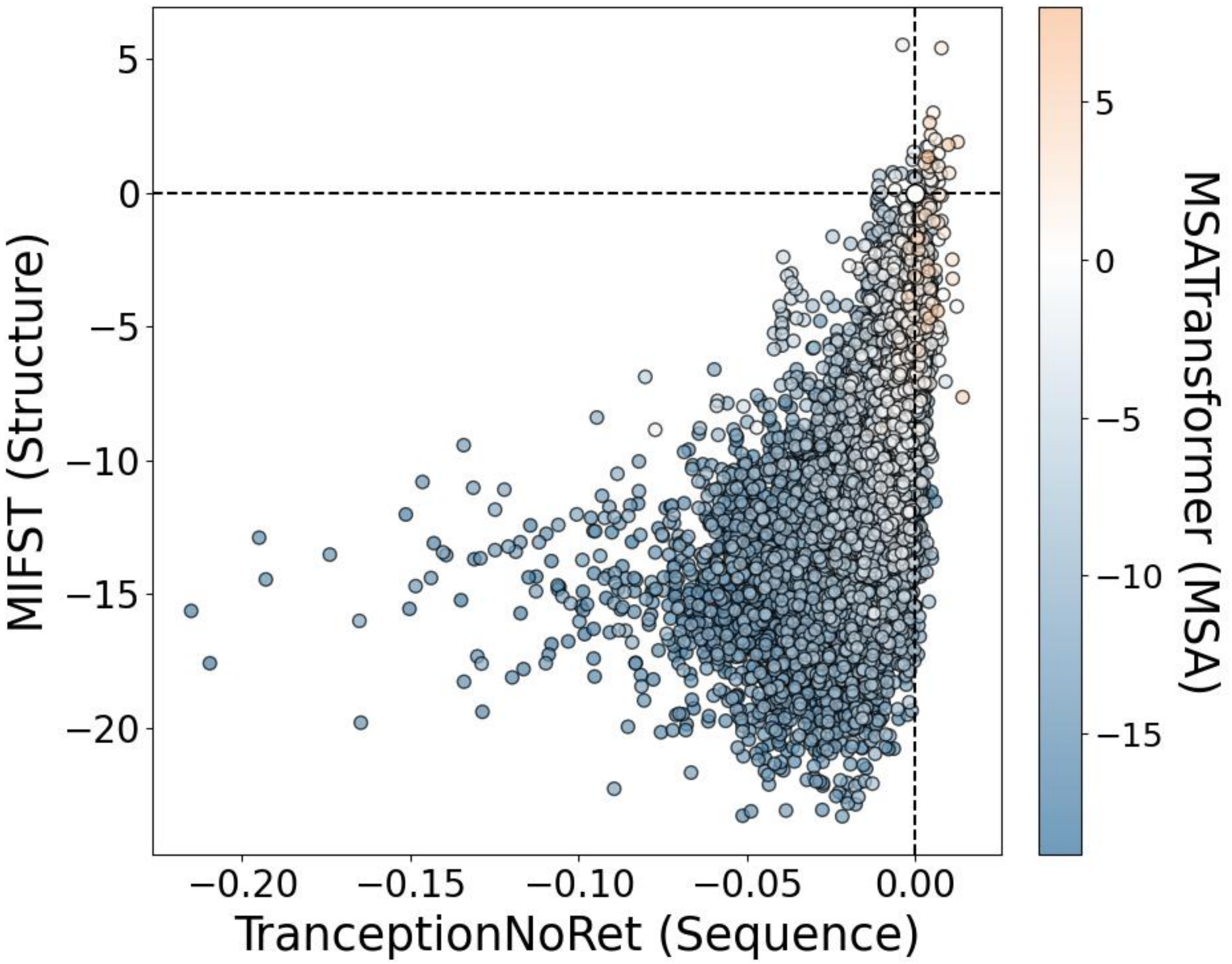

Figure S23 – Zero-shot scores for *in silico* library of *Gm*SuSy. Zero-shot scores for best sequence-based model (Tranception No Retrieval) vs. best structure-based model (MIFST) vs. best MSA-based model (MSA Transformer). Dotted lines and white color denote zero-shot scores for WT, while positive values indicate variants better than WT.

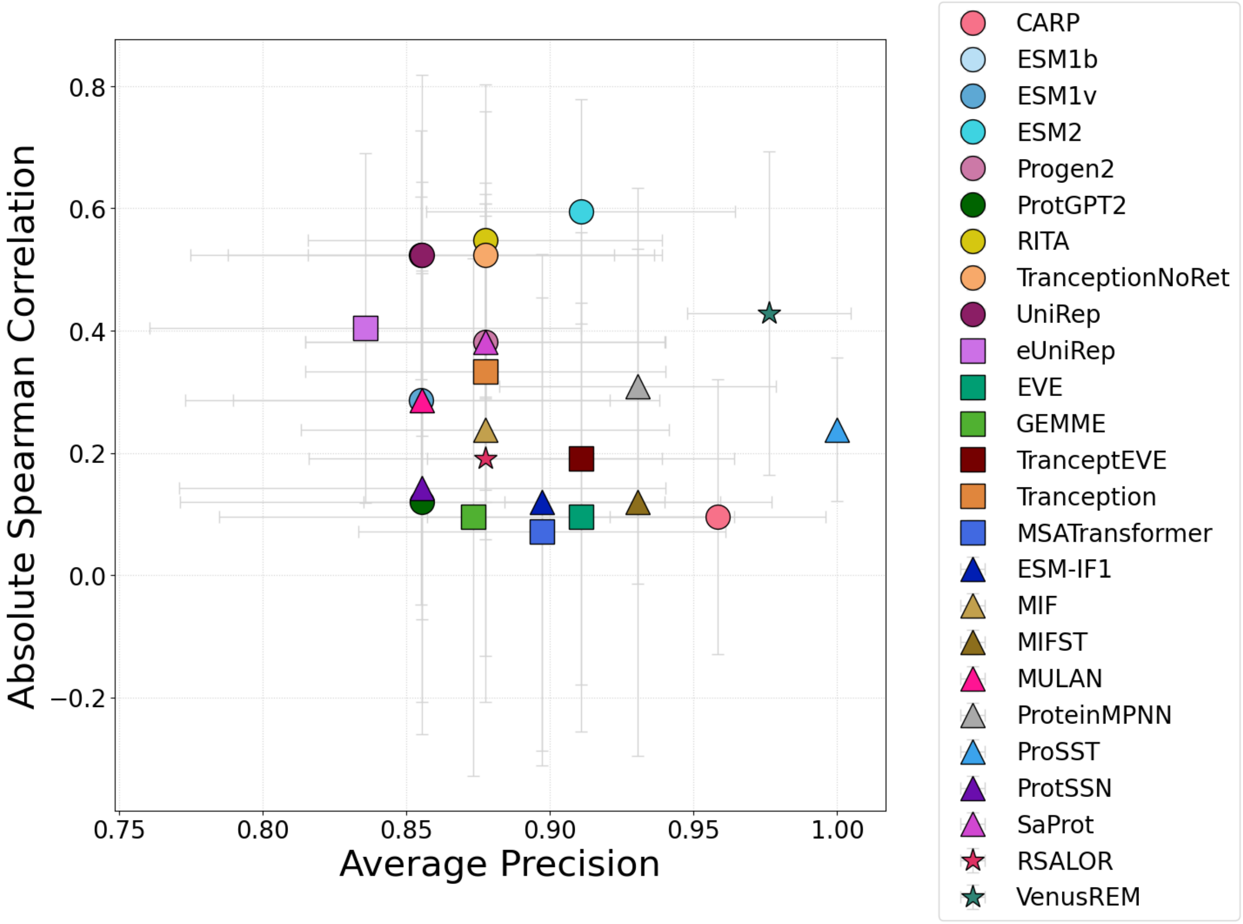

Figure S24 – Performance metrics for the PRIZM zero-shot models when predicting TOGT1_1 relative activity. Circle = sequence-based model, square = MSA-based model, triangle = structure-based model, star = all three. Error bars indicate the estimated standard deviation using a bootstrapping approach (N = 1000).

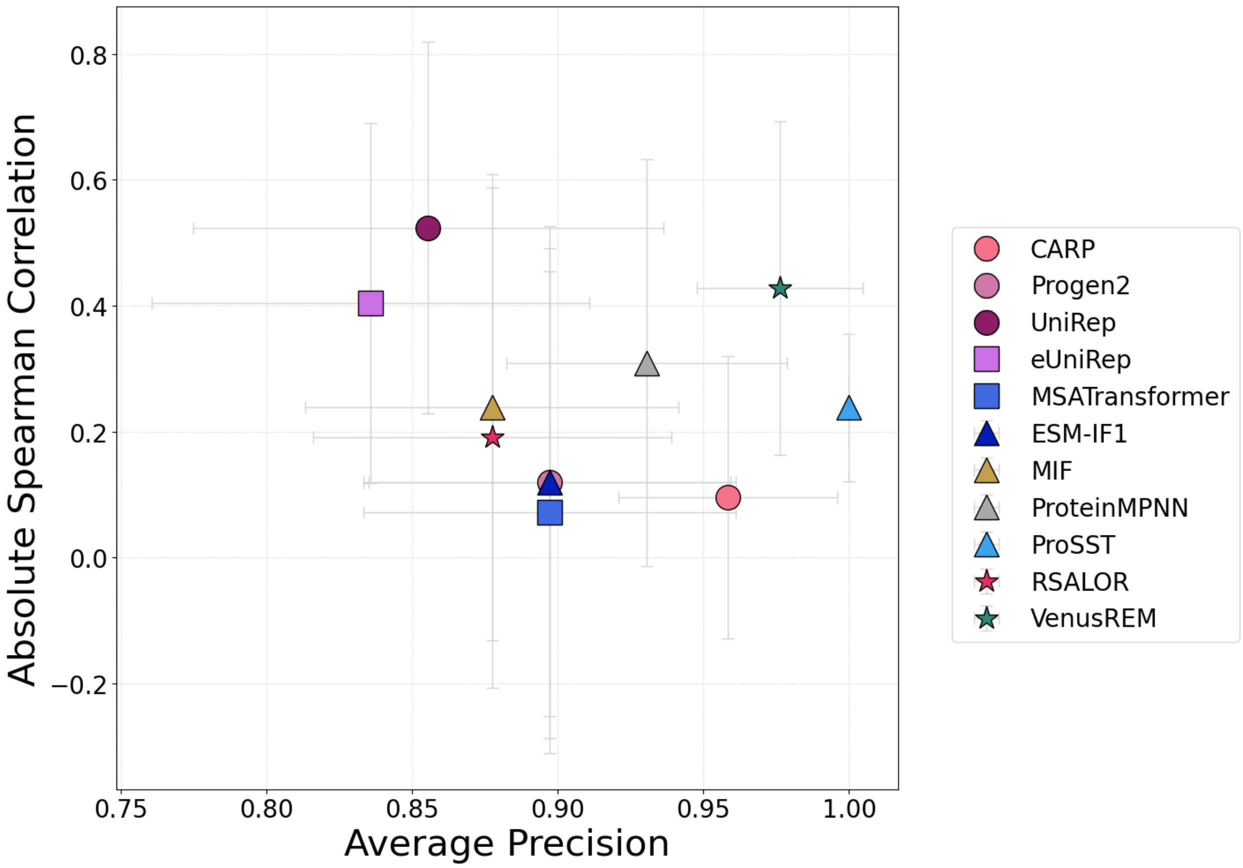

Figure S25 – Performance metrics for the PRIZM zero-shot models with correct direction when predicting TOGT1_1 relative activity. Only models predicting the correct direction of the mutational effect (e.g., higher values corresponding to improved variants) are shown. Circle = sequence-based model, square = MSA-based model, triangle = structure-based model, star = all three. Error bars indicate the estimated standard deviation using a bootstrapping approach (N = 1000).

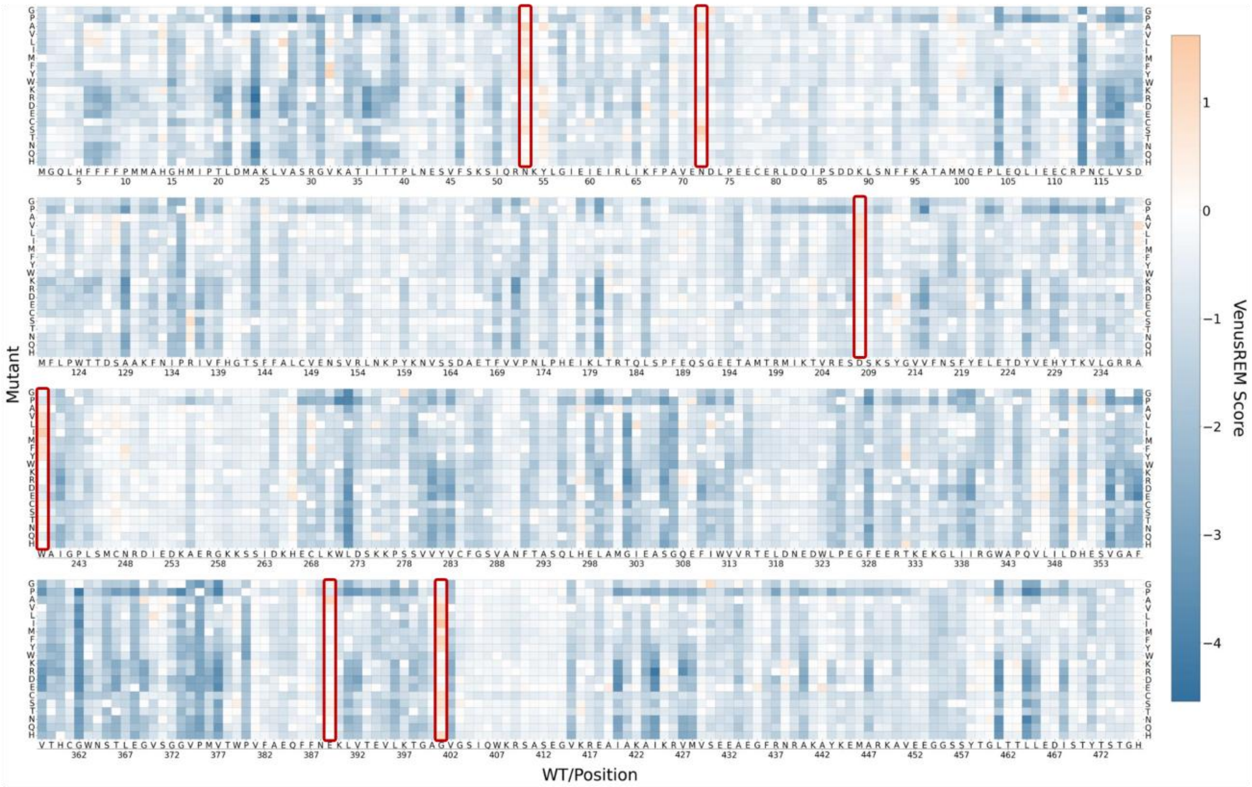

Figure S26 – TOGT1_1 in silico landscape of VenusREM scores. Orange denotes variants predicted to be better than WT, while blue denotes variants predicted to be worse. Red squares mark positions selected for experimental characterization due to high proposed mutability.

Supplementary Tables

Table S1 – DMS benchmark datasets used for validating PRIZM.

**ProteinGym** (Notin *et al.* 2023) **ID**	**Protein**	**Property**	**Reference**
A4_HUMAN_Seuma_2022	Amyloid beta	Aggregation	(Seuma, Lehner, and Bolognesi 2022)
ADRB2_HUMAN_Jones_2020	β_2-adrenergic receptor	Receptor activity (Transcription)	(Jones *et al.* 2020)
ESTA_BACSU_Nutschel_2020	Lipase A	Thermostability	(Nutschel *et al.* 2020)
MK01_HUMAN_Brenan_2016	Mitogen-activated protein kinase 1	Inhibitor resistance	(Brenan *et al.* 2016)
SC6A4_HUMAN_Young_2021	Sodium-dependent serotonin transporter	Fluorescence	(Ellis *et al.* 2024)
SPIKE_SARS2_Starr_2020 _binding	SARS-CoV-2 spike receptor binding domain	Receptor binding	(Starr *et al.* 2020)
YAP1_HUMAN_Araya_2012	Human Yes-associated protein	Peptide binding	(Araya *et al.* 2012)
ANCSZ_Hobbs_2022	Tyrosine kinase	Enzyme activity	(Hobbs *et al.* 2022)
Q59976_STRSQ_Romero_2015	β-glucosidase	Enzyme activity	(Romero, Tran, and Abate 2015)
VKOR1_HUMAN_Chiasson_2020 _activity	Epoxide reductase	Enzyme activity	(Chiasson *et al.* 2020)

Table S2 – Experimental $T_{m,app}$ and residual activity for best *Gm*SuSy variants as predicted by PRIZM.

Variant	PRIZM Rank[a]	$\Delta T_{m,app}$	Residual Activity
WT	N/A	0 ± 0.18	23.4 ± 11.1
Q528M	1	−0.12 ± 0.17	16.7 ± 4.3
V658Y	2	1.45 ± 0.84	3.0 ± 4.3
H534Y	3	−0.21 ± 0.06	26.8 ± 14.1
L731E	4	0.29 ± 0.38	21.1 ± 12.6
F468I	5	2.96 ± 0.20	60.1 ± 19.5

[a] Sequence-wide rank.

Table S3 – Experimental relative activity for selected TOGT1_1 variants.

Variant	PRIZM Rank[a]	Relative Activity
WT	N/A	100 ± 4.28
N53A	2	60.0 ± 4.58
N72S	1	110.5 ± 2.22
W239I	1	102.69 ± 4.71
G401F	3	119.9 ± 2.84
G401I	1	114.1 ± 2.03

[a] Position-wide rank.

References

Araya CL, Fowler DM, Chen W *et al.* A fundamental protein property, thermodynamic stability, revealed solely from large-scale measurements of protein function. *Proceedings of the National Academy of Sciences* 2012;**109**(42):16858–63. https://doi.org/10.1073/PNAS.1209751109.

Brenan L, Andreev A, Cohen O *et al.* Phenotypic Characterization of a Comprehensive Set of MAPK1/ERK2 Missense Mutants. *Cell Rep* 2016;**17**(4):1171–83. https://doi.org/10.1016/J.CELREP.2016.09.061.

Chiasson MA, Rollins NJ, Stephany JJ *et al.* Multiplexed measurement of variant abundance and activity reveals VKOR topology, active site and human variant impact. *Elife* 2020;**9**:1–25. https://doi.org/10.7554/ELIFE.58026.

Ellis HJ, Chan M, Selvam B *et al.* Deep Mutagenesis of a Transporter for Uptake of a Non-Native Substrate Identifies Conformationally Dynamic Regions. *BioRxiv* 4 May 2024:2021.04.19.440442. https://doi.org/10.1101/2021.04.19.440442.

Harding-Larsen D, Madsen CD, Teze D *et al.* GASP: A Pan-Specific Predictor of Family 1 Glycosyltransferase Acceptor Specificity Enabled by a Pipeline for Substrate Feature Generation and Large-Scale Experimental Screening. *ACS Omega* published online 25 Jun. 2024. https://doi.org/10.1021/ACSOMEGA.4C01583.

Hie BL, Shanker VR, Xu D *et al.* Efficient evolution of human antibodies from general protein language models. *Nat Biotechnol* 2024;**42**(2):275–83. https://doi.org/10.1038/S41587-023-01763-2.

Hobbs HT, Shah NH, Shoemaker SR *et al.* Saturation mutagenesis of a predicted ancestral Syk-family kinase. *Protein Science* 2022;**31**(10). https://doi.org/10.1002/PRO.4411.

Hotelling H, Pabst MR. Rank Correlation and Tests of Significance Involving No Assumption of Normality. *Https://DoiOrg/101214/Aoms/1177732543* 1936;**7**(1):29–43. https://doi.org/10.1214/AOMS/1177732543.

Jiang K, Yan Z, Bernardo M Di *et al.* Rapid in silico directed evolution by a protein language model with EVOLVEpro. *Science (1979)* 2025;**387**(6732). https://doi.org/10.1126/SCIENCE.ADR6006.

Jones EM, Lubock NB, Venkatakrishnan AJ *et al.* Structural and functional characterization of G protein-coupled receptors with deep mutational scanning. *Elife* 2020;**9**:1–52. https://doi.org/10.7554/ELIFE.54895.

Lax BM, Escorial García M, Fernandez-Antunez C *et al.* Enzymatic Glucosylation Enhances the Solubility of Niclosamide but Abrogates Its Therapeutic Efficacy. *ACS Omega* published online 26 Jan. 2026. https://doi.org/10.1021/ACSOMEGA.5C12185.

Manzhos T, Ianevych T, Melnyk O. Average precision at cutoff k under random rankings: expectation and variance. *Modern Stochastics: Theory and Applications* 2026;**13**(3):357–74. https://doi.org/10.15559/26-VMSTA298.

Mejia-Otalvaro F, Delima D, Mendonça C *et al.* Stability engineering of sucrose synthase for robust UDP-glucose regeneration. *BioRxiv* 31 Jan. 2026:2026.01.30.702896. https://doi.org/10.64898/2026.01.30.702896.

Notin P, Kollasch A, Ritter D *et al.* ProteinGym: Large-Scale Benchmarks for Protein Fitness Prediction and Design. *Adv Neural Inf Process Syst* 2023;**36**:64331–79.

Nutschel C, Fulton A, Zimmermann O *et al.* Systematically Scrutinizing the Impact of Substitution Sites on Thermostability and Detergent Tolerance for *Bacillus subtilis* Lipase A. *J Chem Inf Model* 2020;**60**(3):1568–84. https://doi.org/10.1021/ACS.JCIM.9B00954.

Romero PA, Tran TM, Abate AR. Dissecting enzyme function with microfluidic-based deep mutational scanning. *Proc Natl Acad Sci U S A* 2015;**112**(23):7159–64. https://doi.org/10.1073/PNAS.1422285112.

Seuma M, Lehner B, Bolognesi B. An atlas of amyloid aggregation: the impact of substitutions, insertions, deletions and truncations on amyloid beta fibril nucleation. *Nat Commun* 2022;**13**(1):1–13. https://doi.org/10.1038/S41467-022-34742-3.

Starr TN, Greaney AJ, Hilton SK *et al.* Deep Mutational Scanning of SARS-CoV-2 Receptor Binding Domain Reveals Constraints on Folding and ACE2 Binding. *Cell* 2020;**182**(5):1295-1310.e20. https://doi.org/10.1016/j.cell.2020.08.012.

Teufel F, Kollasch AW, Huang Y *et al.* Few-shot Protein Fitness Prediction via In-context Learning and Test-time Training. *ArXiv* 2 Dec. 2025. http://arxiv.org/abs/2512.02315 (31 Mar. 2026, date last accessed).

Zhou Z, Zhang L, Yu Y *et al.* Enhancing efficiency of protein language models with minimal wet-lab data through few-shot learning. *Nat Commun* 2024;**15**(1):5566. https://doi.org/10.1038/s41467-024-49798-6.
